# Nanometer Layer Coating to Lactose Particles for Optimization of Dexamethasone Pulmonary Delivery

**DOI:** 10.1002/advs.76929

**Published:** 2026-08-03

**Authors:** Qin Nie, Jiahe Liu, Xiangyu Zhao, Siying Yang, Han Sun, Bingqian Jia, Xiaohong Ren, Xintao Chen, Lixin Sun, Li Wu, Caifen Wang, Jiwen Zhang

**Affiliations:** ^1^ Department of Pharmaceutical Analysis School of Pharmacy Shenyang Pharmaceutical University Shenyang China; ^2^ Center for Drug Delivery Systems Shanghai Institute of Materia Medica Chinese Academy of Sciences Shanghai China; ^3^ University of Chinese Academy of Sciences Beijing China; ^4^ Key Laboratory of Modern Preparation of TCM Ministry of Education Jiangxi University of Chinese Medicine Nanchang China; ^5^ China NMPA Key Laboratory for Quality Research and Evaluation of Pharmaceutical Excipients National Institutes for Food and Drug Control Beijing China

**Keywords:** dexamethasone, dry powder inhalers, lactose, nanometer layer coating, particle engineering

## Abstract

Lactose (LA), the primary carrier in dry powder inhalers (DPIs), often exhibits excessive drug adhesion, which impairs aerosolization. In this study, four coating methods solid mixing, vapor deposition, suspension coating, and solution coating were evaluated to modify LA surfaces. Solution coating with leucine (Leu) proved optimal and was defined as Nanometer Layer Coating (NLC). When blended with jet‐milled dexamethasone (DEX), NLC‐LA increased the fine particle fraction from 20% to 50% and improved flowability, reducing Carr's Index to 24%. Reduced adhesion forces and increased surface smoothness of NLC‐LA were confirmed by scanning electron microscopy, synchrotron radiation micro computed tomography and atomic force microscope. Continuous Leu coverage at nanometer‐scale thickness was verified using Raman imaging, focused ion beam–scanning electron microscopy, time‐of‐flight secondary ion mass spectrometry, and synchrotron radiation x‐ray microdiffraction. In vivo studies demonstrated that DEX DPI formulated with NLC‐LA enhanced bioavailability and allowed a 30‐fold dose reduction while maintaining therapeutic efficacy against high‐altitude acute lung injury. Collectively, the NLC strategy bridges fundamental surface engineering with translational DPI performance, advancing inhalation science and formulation development.

## Graphical abstract

Nanometer layer coating (NLC) was investigated and its mechanism illustrated by advanced characterization techniques. The effects and application for the inhalation of dexamethasone (DEX) were demonstrated through in vitro *and* in vivo evaluation. This approach increased DEX bioavailability and enabled the dose to be reduced to 1/30 of the intravenous dose while maintaining equivalent therapeutic efficacy against high‐altitude acute lung injury (HALI).

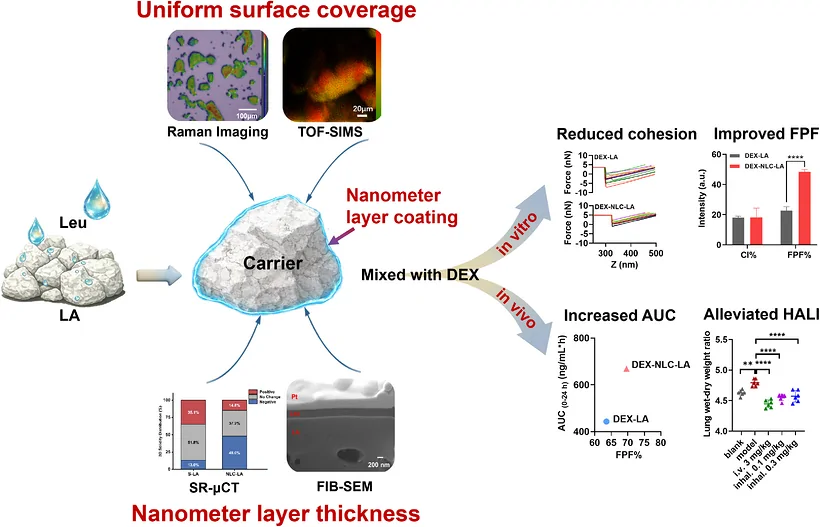

## 1 Introduction

Pulmonary drug delivery using dry powder inhalers (DPIs) has become a clinically mature and widely adopted platform for both local and systemic therapies, owing to its propellant‐free design, formulation stability, portability, and patient‐friendly operation [1, 2, 3, 4, 5]. Among DPI formulation design technologies, carrier‐based formulations remain the most commercially established [6]. Coarse lactose (LA) particles are widely utilized as the dominant carrier excipient in marketed DPI products, serving as a functional “backbone” that enhances powder flow, supports dose uniformity, and enables dispersion of micronized drug particles during inhalation. Generally speaking, LA is cost‐effective, widely available, chemically compatible with many active pharmaceutical ingredients, and supported by extensive regulatory and industrial experience [2, 6]. Therefore, LA‐based carrier systems continue to define the mainstream formulation paradigm for DPIs, making LA carrier engineering a highly impactful route for improving DPI performance and manufacturability. Notably, the physicochemical attributes of LA carriers, including particle size distribution, surface morphology, surface roughness, surface chemistry, and surface energy heterogeneity, strongly influence aerosolization efficiency and lung deposition [5, 6, 7, 8]. Because micronized drug particles rely on controlled detachment from carrier surfaces, LA is not merely an inert diluent but a performance‐determining material that governs both dispersion dynamics and dose reproducibility.

In LA carrier‐based DPI systems, micronized drug particles adhere to the surfaces of LA particles to form interactive mixtures [9]. During inhalation, the therapeutic fraction of the inhaled drug reaching the deep lung largely depends on drug detachment from the carrier under aerodynamic and inertial forces, making controlled de‐adhesion a prerequisite for effective inhalation delivery [10]. Consequently, drug–carrier interactions must be carefully balanced: excessively strong adhesion hinders detachment and lowers the fine particle fraction (FPF), while overly weak interactions can compromise blend uniformity and dose consistency during handling and device actuation [11]. A useful conceptual framework for this dilemma is the cohesive–adhesive balance. Optimal aerosol performance often arises not from minimizing adhesion absolutely, but from tuning the relative magnitudes of drug–drug cohesion and drug–carrier adhesion to enable efficient detachment and deagglomeration under inhalation shear and turbulence [12, 13]. This implies that rational engineering of interfacial forces, rather than empirical adjustment, should be central to new DPI research and development.

Currently, multiple approaches have been proposed to modulate drug–carrier interactions. Classic strategies include: (i) tailoring the particle size and size distributions of LA carrier; (ii) incorporating fine LA particles; (iii) altering the roughness and morphology of LA carrier; and (iv) adding force‐controlling agents (FCAs) such as magnesium stearate (MgSt), leucine (Leu), or other surface‐active excipients [13, 14, 15, 16, 17]. In general, these methods aim to reduce the strong adhesion of drug particles at the critical binding sites of LA, enhance deagglomeration, and improve detachment during inhalation. Among them, FCAs have received sustained attention because they can improve aerosolization performance at relatively low mass fractions, primarily by masking high‐energy adhesion sites on carrier surfaces and reducing drug–carrier interfacial adhesion forces [12, 16, 17]. However, many FCA‐based strategies comprise of simple physical blending that require high additive contents, often ranging from ∼1% up to 5% (w/w) or even higher in some formulations, to achieve consistent performance enhancement [18]. Such approaches frequently result in heterogeneous surface coverage, incomplete masking of high‐energy sites, and poor reproducibility due to the stochastic nature of powder mixing [19]. In addition, the spatial distribution of FCAs on carrier surfaces is difficult to control, leading to batch‐to‐batch variability and formulation‐dependent performance fluctuations [4, 20]. Moreover, owing to the intrinsic complexity of powder systems, improvements in FPF are often reported without sufficient mechanistic characterization linking surface chemistry, surface energy heterogeneity, and measurable interparticle forces to performance outcomes [21, 22]. Consequently, despite extensive empirical success, a gap remains between formulation practice and mechanistic understanding of how FCA distribution, interfacial interactions, and surface architecture collectively govern DPI performance.

In recent years, coating‐based particle engineering has emerged as a promising alternative to conventional blending, offering improved control over LA surface properties through more uniform and reproducible surface coverage [23]. Compared with simple solid particle mixing, coating approaches allow the deliberate deposition of thin layers of lubricants, surfactants, or functional excipients onto carrier surfaces, resulting in better‐defined interfacial modifications. Such controlled surface engineering can effectively tune drug–carrier adhesion and dispersion behavior while minimizing changes to the bulk composition, and preserving the intrinsic flow characteristics of coarse lactose carriers [16, 23, 24]. Representative examples include dry powder coating with magnesium stearate, fluid‐bed or solvent‐mediated coating of amino acids, and spray‐assisted surface modification, all of which have demonstrated improved aerosolization performance compared with conventional physical blending [23, 24, 25]. Moreover, while numerous studies report improved aerosolization, systematic relationships between coating layer thickness, surface coverage intactness, interparticle force profiles, and macroscopic aerodynamic performance (FPF, emitted dose, dose uniformity, flow function) remain insufficiently mapped. Nevertheless, addressing this gap requires integration of advanced surface analysis, interfacial force quantification, and bulk powder rheology with aerosol performance evaluation, to establish a coherent mechanistic pathway from coating architecture to functional DPI outcomes.

In this study, a Nanometer Layer Coating (NLC) strategy was established to enable the simultaneous optimization of aerodynamic performance and rheological properties of DPI formulations. Various coatings were systematically evaluated and compared in terms of their impacts on LA surface roughness, drug‐carrier adhesion, and aerosolization behaviors, using jet‐milled dexamethasone (DEX) as a model drug to form DEX‐NLC‐LA particles, defined as DEX DPI. The underlying mechanisms were elucidated through multi‐scale characterization, including scanning electron microscopy (SEM), atomic force microscope (AFM), synchrotron radiation micro computed tomography (SR‐µCT), synchrotron radiation x‐ray microdiffraction (SR‐µXRD) and focused ion beam–scanning electron microscopy (FIB‐SEM). Meanwhile, pharmacokinetic and high‐altitude acute lung injury (HALI) evaluations of inhaling DEX DPI in rats were performed. Collectively, this study established NLC as a rational and versatile particle engineering strategy for DPI carriers and demonstrated that nanometer‐scale surface modification of LA can markedly enhance both pulmonary deposition and powder flowability of DEX, thereby providing a simple yet effective approach for the development and optimization of carrier‐based DPI formulations (Scheme 1).

**SCHEME 1:**
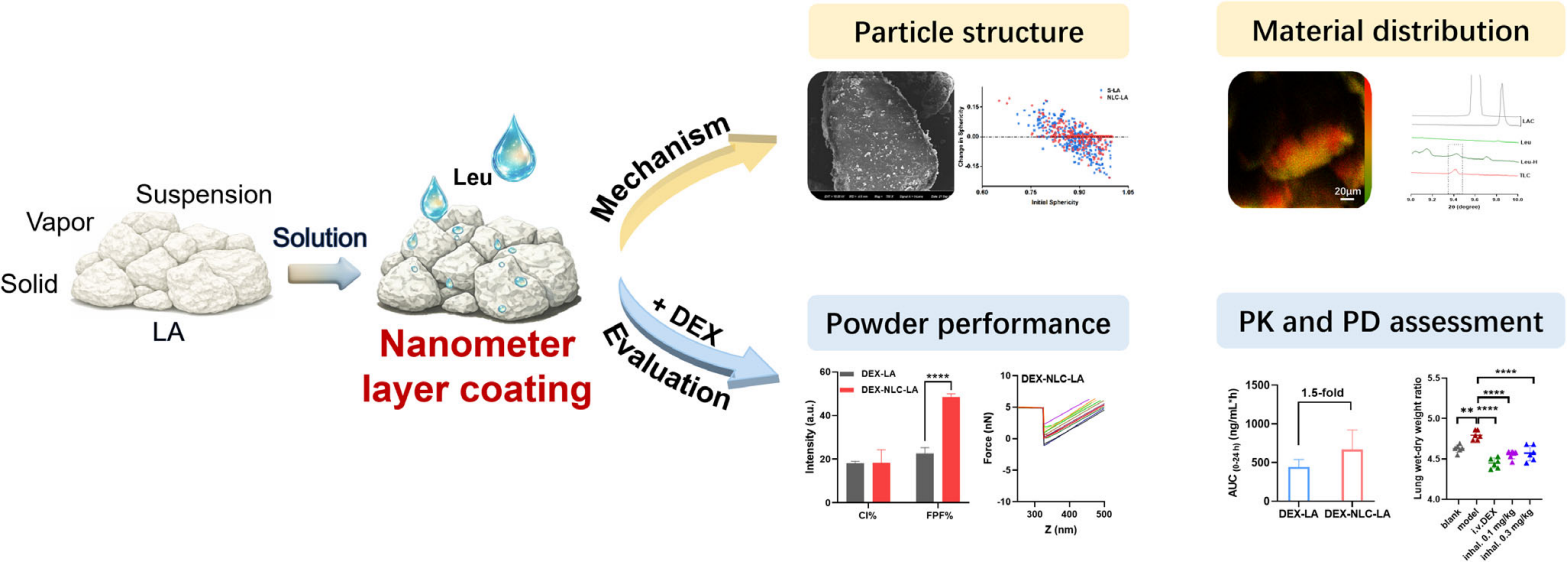
Screening strategy for nanometer layer coatings, mechanism investigation, and efficacy study of optimal DEX DPI formulations.

## 2 Materials and Methods

### 2.1 Materials

Dexamethasone (DEX, > 99%) was purchased from Meilun Pharmaceutical Co., Ltd. (Dalian, China). Respitose SV001, Respitose SV010, Lactohale LH200, and Respitose ML003, were obtained from DFE Pharma (Germany). Leucine and 5‐hydroxymethylfurfural (5‐HMF, > 98%) were provided by Shanghai Titan Technology Co., Ltd. (Shanghai, China) and J&K Scientific Ltd. (Beijing, China) respectively. Magnesium stearate (MgSt) was purchased from Fengli Jingqiu Pharmaceutical Co., Ltd. (Beijing, China). Glycerol (Gly), Poloxamer 407 (P407), Tween 20 (T20), Tween 80 (T80), Oleic acid (OA), Liquid paraffin (LP), Span 80 (S80), Arginine (Arg), Tyrosine (Tyr), Cholesterol (Cho), Stearyl alcohol (SA), Paraffin (PA), and Stearic acid (SAc) were purchased from Sinopharm Chemical Reagent Co., Ltd. (Shanghai, China). Borneol (Bo), linalool (Li) and menthol (Me) were obtained from Jiangxi Linyuan Fragrance Co., Ltd. (Jiangxi, China), TCI Chemical Industry Development Co., Ltd. (Shanghai, China), and BASF SE (Ludwigshafen, Germany), respectively.

Healthy male Sprague‐Dawley (SD) rats (250 ± 20 g) were supplied by Shanghai Lab Animal Research Center (Shanghai, China). The animals were housed in a temperature (22°C ± 1°C) and humidity (65%–70%) controlled room with a 12 h light‐dark cycle, and had free access to food and water. All animal experiment protocols were approved by the Animal Care and Use Committee of Shanghai Institute of Materia Medica, Chinese Academy of Sciences (IACUC Application No. 2025‐02‐ZJW‐55) and conducted in accordance with the National Research Council's Guide for the Care and Use of Laboratory Animals.

### 2.2 Surface Modification of LA Carriers

#### 2.2.1 Vapor Coating

The surface modifier (Bo, Me, and Li) was mixed with LA (SV010) at a mass ratio of 0.1: 99.9 (w/w) and agitated at 40 rpm for 30 min to form powder mixture, which was then placed in a centrifuge tube (50 mL), incubated in a water bath at 70°C, and magnetically stirred at 400 rpm for 30 min to facilitate the vaporization of the modifiers, before it is subsequently cooled down to let the vaporized agents deposit onto the surface of the LA particles. Finally, after incubation at 50°C for 2 h to eliminate excess steam, the vapor‐coated LA powder was obtained.

#### 2.2.2 Suspension Coating

LA was placed in a centrifuge tube (50 mL), incubated in a 50°C water bath, and magnetically stirred at 400 rpm. The surface modifiers (Leu, MgSt, Arg, Tyr, Cho) were dispersed in anhydrous ethanol to form suspension (20 mg/mL), and aliquots of 50 µL were added dropwise to the LA (modifier ratio: 0.1% of LA). After continuous stirring for 30 min, the mixture was dried in a forced‐air drying oven (Shanghai YIHENG Technical Co., Ltd) at 50°C for 2 h to obtain the suspension‐coated LA powder.

#### 2.2.3 Solution Coating

LA was placed in a centrifuge tube (50 mL) and incubated in a 50°C water bath while stirring at 400 rpm. The surface modifiers (Leu, LP, T80, T20, Gly, P407, S80, and OA) were dissolved in anhydrous ethanol to form solution (20 mg/mL), and aliquots of 50 µL were added dropwise to LA (modifier ratio: 0.1% of LA). Leu was first dissolved in 1 m HCl solution, noted as Leu‐H. After continuous stirring for 30 min, the mixture was dried at 50°C in a forced‐air drying oven for 2 h to obtain the solution‐coated LA powder.

#### 2.2.4 Solid Coating

LA was placed in a round‐bottom centrifuge tube (50 mL), incubated in a 70°C water bath, and magnetically stirred at 400 rpm. SAl, PA, and SAc were selected and added as surface modifiers into LA at a mass ratio of 0.1: 99.9, followed by magnetic stirring for 2 h. The product was then dried at 50°C in a forced‐air drying oven for 2 h to obtain the coated LA powder.

### 2.3 Optimization of LC Conditions

The solution coating process was optimized by screening for Leu content, addition volume (ethonal), and treatment cycles. The Leu content was set at 0.001%, 0.005%, 0.01%, 0.05%, 0.10%, 0.20% (w/w), with 0.10% corresponding to a Leu: LA mass ratio of 0.001: 1 g (w/w). Next, Leu‐H ethanol solutions with different concentrations (0.5, 1, 2, 5 mg/mL) were prepared, and 20, 50, 100, and 200 µL of the solutions were added to LA (Leu ratio: 0.01% of LA) to optimize the solvent dosage. Then, LA (1 g) was coated with 50 µL of 10 mg/mL Leu‐H ethanol solution (0.05% dosage, 50 µL/g), stirred for 30 min, and dried in forced‐air drying oven at 50°C for 2 h. The operation was repeated two or three times. Furthermore, various types of LA (SV001, LH200, and ML003) were treated with Leu solution (20 mg/mL, modifier ratio: 0.1% of LA) to examine the coating effect. All layer coating LA particles were defined as LC‐LA.

### 2.4 Reproducibility and Stability of Optimal Processes

LA (2.5 g) was weighed and transferred into a 100 mL centrifuge tube, with the latter being placed in a 50°C water bath with stirring at 400 rpm. LA was coated with 250 µL of 5 mg/mL Leu‐H ethanol solution (0.05% dosage), stirred for 30 min, and dried in forced‐air drying oven at 50°C for 2 h to obtain NLC‐LA. A total of six parallel batches were prepared under identical conditions. The NLC‐LA powder was placed for 6 months and then examined its stability under a temperature of 25°C and a humidity of 45%. In addition, the residual ethanol, pH, and degradation formation (5‐HMF) of NLC‐LA were analyzed, and the determination methods were detailed as “*Determination of residual solvent and degradation formation*” section (Supplementary Material).

### 2.5 Preparation and Evaluation of DEX DPIs

Jet‐milled DEX powders as model drug were prepared using the facility provided by Shanghai Nuoze Fluid Technology Co., Ltd. (Shanghai, China). All LC‐LA particles were mixed with DEX, which was added using equivalent incremental method at a ratio (LA: DEX = 98: 2, w/w) [26], vortexed at 1500 rpm for 30 min to obtain the powder, then passed through a 100‐mesh sieve, and subsequently dried at 50°C in forced‐air drying oven for 2 h to obtain DEX‐LC‐LA powders. Meanwhile, unmodified LA was mixed with DEX using the same method as a control, defined as DEX‐LA. Additionally, Leu (0.1% and 0.2% of LA) was mixed with LA particles to obtain physical mixture (PM), which was then blended with DEX, defined as DEX‐PM. The preparation was carried out at room temperature with humidity below 50% RH. The aerodynamic behaviors of DEX DPIs were evaluated using the Next Generation Impactor (NGI) (Copley Science Ltd., Nottingham, United Kingdom). The determination method was detailed as “*NGI tests*” section (Supplementary Material). The flowability of all the powders was evaluated using the Carr's Index (CI), calculated from bulk and tapped density. The methodology followed a previously described protocol [27] with modification, as detailed in “*Carr's Index*” of the Supplementary Material.

### 2.6 Particle Structure of NLC‐LA

#### 2.6.1 SEM

The appearance of LA, NLC‐LA, and PM was investigated using SEM (ZEISS Gemini 300, Carl Zeiss AG). Samples were mounted on double‐sided conductive carbon tape and sputter‐coated with gold. The SEM images were captured at an accelerating voltage (EHT) of 2.00 kV, a working distance (WD) of 4.9 mm, and a magnification of 700 ×.

#### 2.6.2 SR‐µCT

SR‐µCT was conducted at the BL13HB beamline of the Shanghai Synchrotron Radiation Facility (SSRF) to characterize the 3D structures of LA, solvent‐coated LA (S‐LA), and NLC‐LA particles. Imaging was performed at a photon energy of 22 keV with an effective pixel size of 3.25 µm. Phase‐contrast retrieval and 3D reconstruction were carried out using PITRE (v3.1), followed by visualization and quantitative analysis in Avizo (Thermo Fisher Scientific). Particle‐level morphological parameters, including volume, sphericity, and 3D solidity, were quantified. Pre‐ and post‐treatment particles were matched one‐to‐one to assess morphology changes induced by solvent and Leu‐H treatment, with variations below 1 × 10^−6^ considered negligible. Detailed preparation procedures, acquisition settings, reconstruction preprocessing, and the particle‐matching and statistical workflow were provided in SM‐CT1 to SM‐CT3 (Supplementary Material). Furthermore, the coating thickness of the NLC‐LA particles was calculated based on the total surface area (A_total_) and total volume (V_total_), which was detailed as “*Calculation of the coating thickness of NLC‐LA particles*” section (Supplementary Material).

#### 2.6.3 FIB‐SEM

The coating thickness of the NLC‐LA particles observed using FIB‐SEM (ZEISS Crossbeam 540, Germany) (Supplementary Material). Individual NLC‐LA particles were selected and imaged by SEM at an accelerating voltage of 3 kV, probe current of 800 pA, and a working distance of 5.0 mm. A protective platinum layer was deposited prior to FIB processing. Cross‐sectioning was performed using a Ga^+^ ion beam at 30 kV and 100 pA, followed by SEM imaging of the milled cross‐sections at a tilt angle of 54° to obtain high‐resolution cross‐sectional images.

### 2.7 Particle Interaction Between NLC‐LA and DEX

Surface roughness of NLC‐LA was also measured using a Bruker Dimension Icon AFM (Bruker Corporation, USA) in Tapping mode. All samples were dispersed on Teflon double‐sided tape, and a PMCL AC 240TS probe was used. The scanning range was 20 µm, scanning rate was 0.4–0.5 Hz, and integral gain was 345 ± 20 mV. The root mean square roughness (Rq) were calculated using Nanoscope software. The adhesion force of NLC‐LA carriers and DEX was measured using AFM as: DEX particle probes were prepared using epoxy resin AB glue (1:1, w/w). The probes were calibrated for deflection sensitivity and spring constant using a mica sheet. The adhesion force between DEX and LA was measured in contact mode, and 12 positions were tested for each sample. The adhesion force was calculated as the absolute value of the difference between the baseline ordinate (Y_0_) and the minimum ordinate (Y_1_) in the separation stage of the force curve.

### 2.8 Raman Spectroscopy and Chemical Imaging of NLC‐LA

Standard Raman spectra of pure Leu and LA were acquired using a DXR3xi Raman Imaging Microscope (Thermo Fisher Scientific) to serve as references for subsequent correlation analysis. The acquisition parameters were set as follows: excitation wavelength of 532 nm, laser power of 10 mW, exposure time of 0.05 s with 10 accumulations, and a spectral range of 50–3400 cm^−1^. Raman mapping was conducted to visualize the spatial distribution of Leu on the surface of LA‐based particles, including LA, NLC‐LA, and PM samples. The mapping parameters were identical to those used for reference spectra, with a step size of 5 µm. Correlation analysis based on the Leu reference spectrum was applied to generate pseudo‐color maps of correlation coefficients (r), where red and green represent high and low similarity, respectively. The color scale was adjusted to 0.25–0.55 to enhance visualization of Leu enrichment and distribution on the LA matrix surface.

### 2.9 Surface Material Distribution of NLC‐LA

The time‐of‐flight secondary ion mass spectrometry (TOF‐SIMS) is a surface‐sensitive mass spectrometry technique used to characterize the elemental composition of the outermost atomic layers of materials, which operates by bombarding the sample surface with a focused high‐energy ion beam, inducing the emission of secondary ions that are analyzed by TOF‐SIMS to determine their mass‐to‐charge (*m/z*) ratios [28, 29]. It provides high analytical sensitivity (down to 10^6^ atoms/cm^2^) and high spatial resolution (∼0.2 µm), making it particularly suitable for probing elemental distribution and thin coating layers on particle surfaces [29, 30].

The surface composition and material distribution of samples (LA, NLC‐LA and PM) were analyzed using a SurfaceSeer‐I TOF‐SIMS (KORE Technology, UK) operated in positive ion mode. Samples were spread on double‐sided conductive tape, and the ion beam energy was 30 kV. The characteristic peaks of LA ([M+Na]^+^ = *m/z* 365) and Leu ([M+H]^+^ = *m/z* 132) were detected, and the scanning range was 200 × 200 µm. The distribution images were merged using Image J software.

### 2.10 Crystal Morphology of Coating Layer

SR‐µXRD experiments were conducted at the BL17UM‐High Performance Membrane Protein Crystallography Beamline of the Shanghai Synchrotron Radiation Facility to assess the coating condition of LA by identifying the diffraction peaks of Leu, which employed x‐rays with an energy of 19.97 keV (wavelength = 0.0638588 nm) and a spot diameter of approximately 10 µm [31]. All powder samples (LA, Leu, Leu‐H, NLC‐LA, and PM) were mounted on Compton film and scanned by translating the sample stage across the x‐ray beam. Single‐point and 2D scans were conducted in an S‐shaped pattern with particle‐size–dependent step sizes and a 5 s exposure time. For NLC‐LA samples with low Leu content, localized 2D scans were performed. Diffraction signals from LA and PM were collected simultaneously for comparison, and background signals arising from graphite were subtracted during data processing [32]. The sample‐to‐detector distance was calibrated using a CeO_2_ standard with Dioptas software, and conventional XRD patterns were obtained by integrating the 2D diffraction images.

### 2.11 Pharmacokinetic Evaluation of DEX‐NLC‐LA

The pharmacokinetic experiments were conducted using the aerosol oral and nasal inhalation drug delivery system (MELTON, Shanghai Hanfei Medical Devices Co., Ltd., Shanghai, China). Six rats were randomly allocated into two treatment groups (*n* = 3) to receive either inhaled DEX‐LA or inhaled DEX‐NLC‐LA, both administered at a dose of 3.0 mg/kg (calculated as DEX). Animals were fasted for 12 h before the experiment until 4 h after dosing, with free access to water. Prior to the drug administration, blank plasma was collected and 50 mg/mL activated charcoal suspension in normal saline was administered by intragastric gavage to reduce gastrointestinal absorption. Blood samples (0.3 mL) were collected into heparinized centrifuge tubes at 5, 10, 15, 30, 45 min, and 1, 2, 4, 6, 10, and 24 h post‐dosing. The samples were then centrifuged at 3000 rpm for 10 min to separate plasma. The concentration of DEX in rat plasma was determined using a previously reported method [33], as detailed in the “*Concentration determination of plasma*” section (Supplementary Material).

### 2.12 Efficacy of DEX‐NLC‐LA in HALI Model

Rats (*n* = 36) were randomly allocated into six experimental groups (*n* = 6); the blank group, HALI model group, intravenous injection group (i.v. DEX, 3 mg/kg, as the positive control), inhal. DEX‐NLC‐LA group (high‐dose, 0.3 mg/kg), and inhal. DEX‐NLC‐LA group (low‐dose, 0.1 mg/kg). All treatment groups received two pre‐treatment doses. Following the second administration, the model group and all treatment groups received an intravenous injection of Lipopolysaccharide (LPS, 0.5 mg/kg) to induce HALI. Rats were immediately placed in a low‐pressure chamber (Yuyan Instruments Co., Ltd., Shanghai, China) simulating a high‐altitude environment (5000 m) to established the HALI model, while the blank group remained in ambient oxygen conditions. After 4 h, the chamber pressure was restored to atmospheric pressure, and rats were immediately anesthetized and dissected. During sample collection, blood was obtained for routine hematological parameter testing. Fresh lung tissue was fixed at the same height to document lung morphology. Left lung tissue was used for wet‐dry weight ratio determination; the right lung upper lobe was fixed in paraformaldehyde solution for hematoxylin and eosin (H&E) staining to assess injury severity, while the right lung lower lobe was reserved for ELISA assays to measure the levels of the inflammatory factors, including tumor necrosis factor‐alpha (TNF‐α), interleukin‐1 beta (IL‐1β), and interleukin‐6 (IL‐6) (HUABIO, Youke Life, Hangzhou, China).

### 2.13 Statistical Analysis

The pharmacokinetic parameters were calculated using DAS 2.0 software. The statistical analyses were expressed as mean ± standard deviation (SD) or relative standard deviation (RSD) and performed using GraphPad Prism 8 (San Diego, CA, USA) and Origin. Statistical significance was defined using one‐way analysis of variance (ANOVA) and *t*‐test. The values of ^*^ *p* < 0.05, ^**^ *p* < 0.01, ^***^ *p* < 0.001, ^****^ *p* < 0.0001 and *ns* (no significant) were applied to annotate statistical significance.

## 3 Results

### 3.1 Screening of Optimal NLC Process

The CI and FPF of pure DEX were 45.87% ± 0.23% and 68.87%, respectively, confirming its poor flowability [34]. While the addition of unmodified LA improved the flowability of the DEX‐LA blend, it also drastically reduced the FPF by approximately 50%. Most of this powder (67.43%) deposited in the throat and pre‐separator (Table S1). Therefore, different coating methods were investigated to modify LA as a carrier, aiming to improve powder flowability while enhancing FPF. For the vapor coating, the FPF of DEX‐LC‐LA powder was improved using the modified LA as the carrier, which Bo‐coated LA showed the best performance, with percentage of throat and pre‐separation of 52.52% and an FPF of 36.91% (Figure 1A and Table S1). In the suspension coating, all the evaluated modifiers (Leu, LP, T80, etc.) improved the FPF of DEX‐LC‐LA by about 10% in comparison to DEX‐LA, meanwhile, LA treated with solvents alone showed no significant improvement. The most pronounced result was achieved using the solution‐coating approach. When Leu‐H was employed as surface modifier, the resulting DEX‐LC‐LA exhibited a high FPF (39.59%) with an excellent device emptying rate (100.9 ± 0.53%) (Figure 1A and Table S1). Furthermore, the percentage content of throat and pre‐separation decreased from 67.43% to 51.86%, showing that DEX and LA could effectively separate in the airway and thus reach the deep lung (Figure 1A and Table S1). For the solid coating, compared to the DEX‐LA, the FPF of powders had not shown improvement (Figure 1A and Table S1). Additionally, when solid‐state Leu was used to handle LA and mixed with DEX to obtain DEX‐PM particles, the results revealed that even with the introduction of 2% Leu, the FPF of DEX only reached 32%, indicating that this treatment method was not significantly effective (Figure S1A).

**FIGURE 1:**
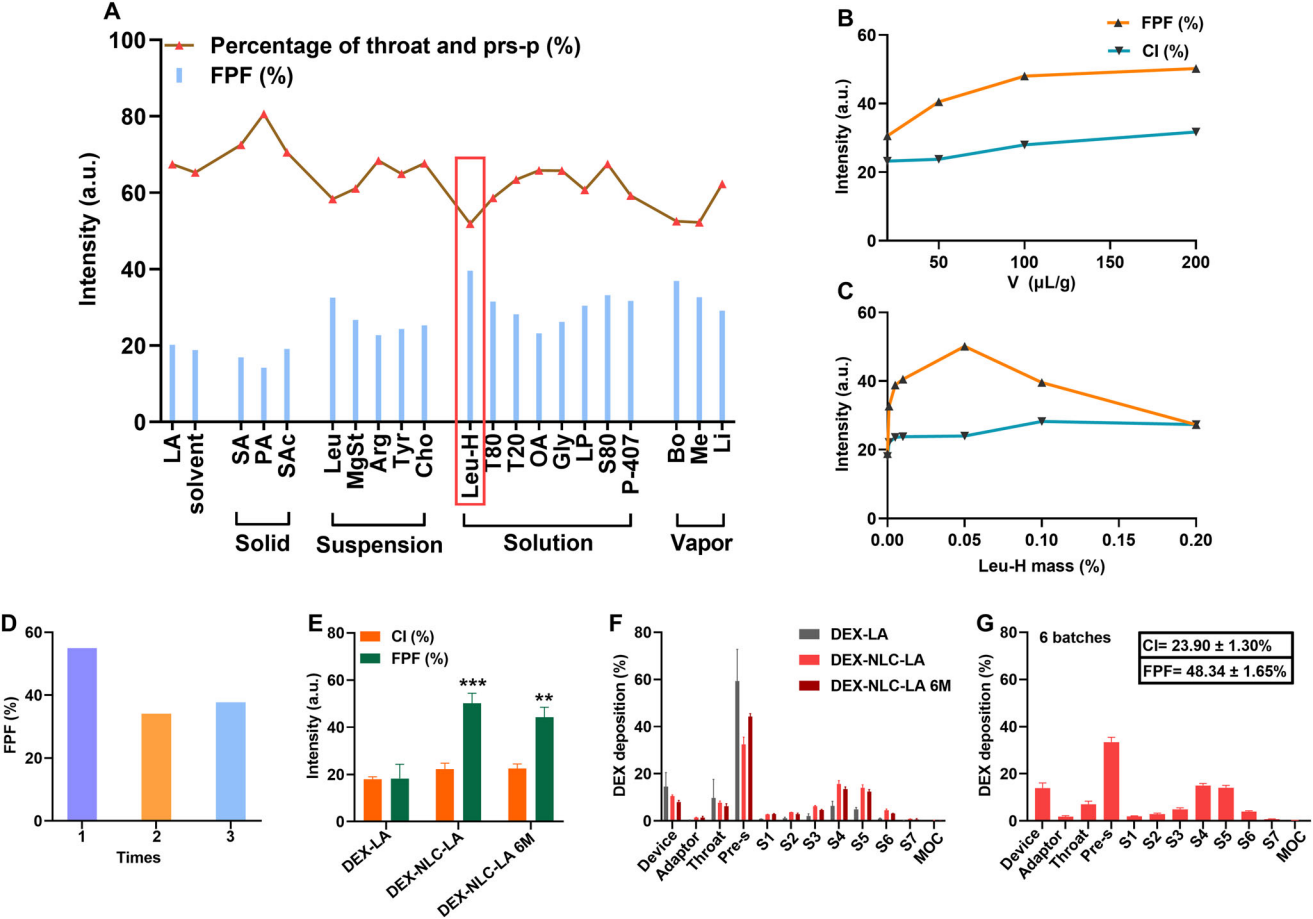
Nanometer layer coating process screening. (A) Comparison of total percentage at the throat and pre‐separator and FPF values after mixing with DEX using the four LA modified coating processes (*n* = 1). Comparison of FPF values of DEX DPIs prepared by blending DEX with LA coated under varying solvent (ethanol) volumes (B), Leu masses (C), and treatment times (D). (E, F) Comparison of aerodynamic parameters and NGI deposition among DEX‐LA, DEX‐NLC‐LA and DEX‐NLC‐LA 6 M (six months) (*n* = 3). (G) DEX‐NLC‐LA deposition of six batches in NGI (*n* = 6). ^*^Compared with DEX‐LA, ^**^p< 0.01, ^***^p< 0.001.

Therefore, Leu‐H was selected as the optimal modifier, and its content, solvent volume, and processing frequency were then investigated to determine the optimal solution coating process. With the increase of solvent volume at a fixed Leu mass (0.01% of LA mass), the FPF of DEX‐LC‐LA powder increased from 30.55% to 50.22%, while the CI increased from 23.23% to 31.73%, indicating reduced flowability (Figure 1B and Figure S1B). The FPF increased with the increase of Leu mass in the range of 0.001%–0.50%, reaching the maximum FPF value of 50.15% at 0.05% Leu mass. When the Leu mass exceeded 0.10%, the FPF was decreased and Leu mass was 0.20% with FPF of 28.79% (Figure 1C and Figure S1C). The FPF of DEX‐LC‐LA powder prepared with LA modified once (50.15%) was higher than that of LA modified twice (30.08%) and three times (37.72%), demonstrating that multiple modifications led to a decrease in FPF (Figure 1D Figure S1D). Following modification with Leu‐H solution, the FPF of DEX DPIs prepared using different types of LA increased by over 10%, which DEX‐LC‐LA formulations using ML003, LH200, SV010, and SV001 as carriers exhibited FPF values of 52.74%, 46.34%, 39.59%, and 23.43%, respectively, indicating markedly improved aerodynamic performance compared with unmodified LA, while flowability remained essentially unchanged (Figure S1E,F).

Through a series of screenings, it was determined that the optimal condition was a single solution‐coating cycle using 0.05% (w/w, relative to LA) Leu‐H with 50 µL of coating solution, forming NLC, which was mixed with DEX to obtain DEX‐NLC‐LA. Compared to DEX‐LA, it exhibited superior aerodynamic behavior (FPF = 50.15%) without significant alteration in flowability (CI = 23.98%). Additionally, the long‐term stability results of DEX‐NLC‐LA under room‐temperature conditions (25°C/40% RH) for 6 months showed that FPF and CI were 44.21% and 22.53%, indicating the DPI process was stable (Figure 1E,F). The content of DEX DPI was 1.99% (RSD = 1.43%), showing that the content of this DPI formulation was relatively uniform. On the other hand, six batches NLC‐LA were prepared under optimal conditions in parallel to examine their reproducibility, the results of which showed that the FPF and CI were 48.34% (RSD = 3.42%) and 23.90% (RSD = 4.84%) respectively, indicating the reproducibility was excellent (Figure 1G).

Considering that Leu‐H may lead to the production of 5‐HMF in LA, a further assessment was conducted on the 5‐HMF content of the newly prepared and stored LC‐LA for 6 months. The results indicated that the 5‐HMF concentration in unmodified LA was 0.06 µg/mL, while that in LC‐LA ranged from 1.38 to 1.70 µg/mL. Additionally, a new batch of LC‐LA was prepared to assess its 5‐HMF content, and the results showed that the 5‐HMF content was 0.43 µg/mL (Table S2). Although the 5‐HMF content in LC‐LA increased slightly over time, it remained within the acceptable limits. And the ethanol content of LC‐LA obtained with different volumes was determined to be 0.47% (4700 ppm) (Table S3), which was below the 5000 ppm (0.5%) threshold specified for relative guidelines [35]. All in all, those results indicated the DEX‐NLC‐LA (defined as DEX DPI) could be used for subsequent experiments.

### 3.2 Particle Surface Roughness and Particle Interaction Between NLC‐LA and DEX

The morphological variations of NLC‐LA were investigated using SEM. LA and PM powder showed a rough and irregular surface covered with many fine particles. In contrast, the NLC‐LA particles displayed a significantly smoother surface, indicating that the Leu coating improved the surface smoothness of LA (Figure 2A).

**FIGURE 2:**
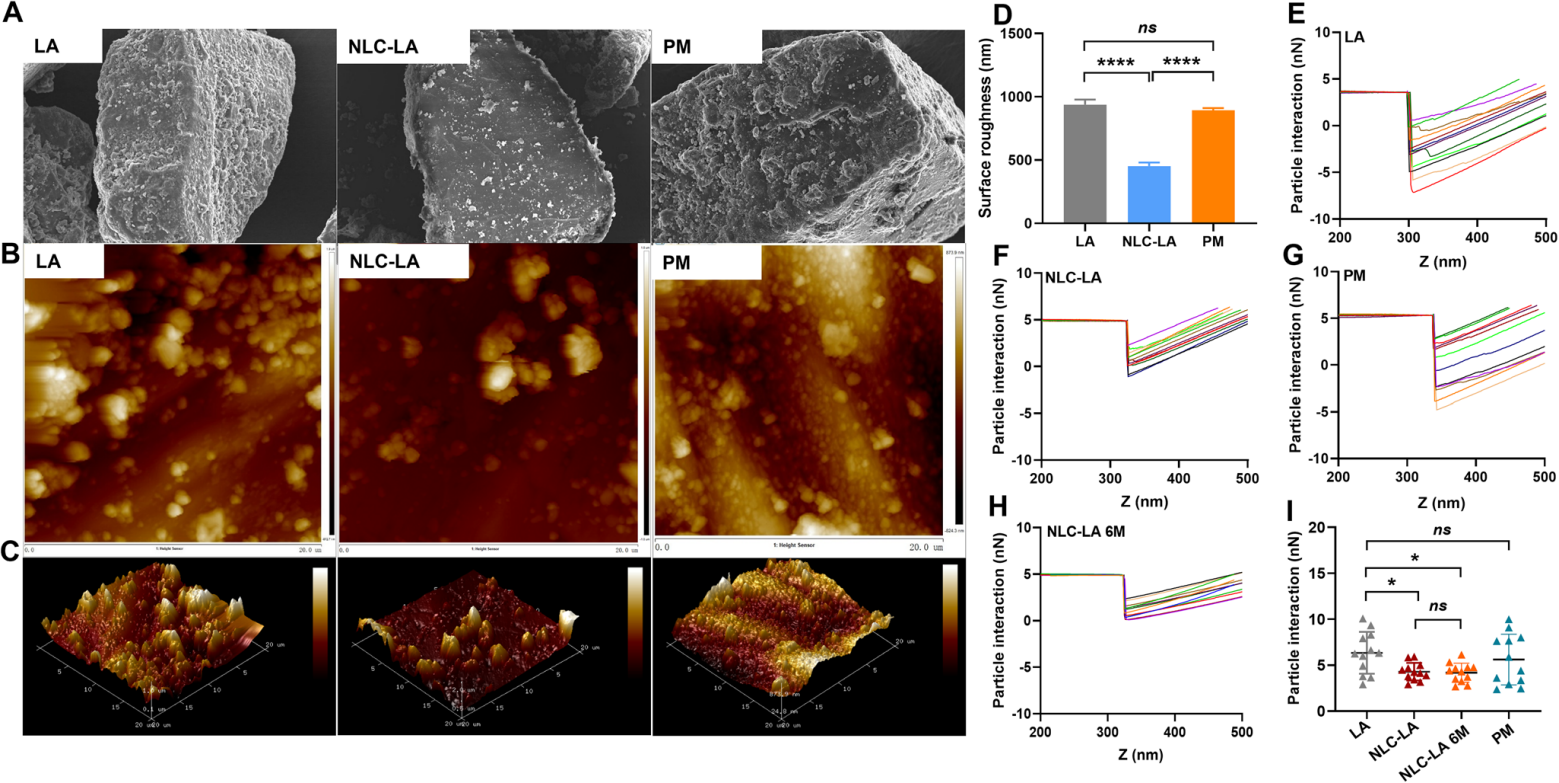
Characterization of appearance and surface roughness of LA, NLC‐LA and PM. (A) SEM images (scale bar = 10 µm). (B, C) The 2D and 3D images of AFM. (D) Comparison of the surface roughness among three powders (*n* = 3). (E–G) Particle interaction force curves between DEX and three types powders (*n* = 12). (H) Particle interaction force curves between DEX and NLC‐LA powders after six months (*n* = 12). (I) Comparison of interaction force between DEX and various powders (*n* = 12). ^*^ *p* < 0.05, ^****^ *p* < 0.0001, *ns* = no significant. *Note*: the content of Leu was 0.1%.

The particle surfaces and roughness of NLC‐LA were further observed using AFM. Numerous bright white “island‐like” protrusions were observed on both LA and PM particles, the corresponding 3D images presented a 3D landscape of alternating peaks and valleys. However, AFM images of NLC‐LA particles exhibited distinctly different characteristics: 2D images showed almost no prominent bright spots, while 3D surfaces displayed only faint, low‐height ripples (Figure 2B,C). Additionally, calculating the Rq for regions of equal particle size across the three powders yielded the following results: with LA (938.7 ± 40.08 nm), NLC‐LA (453.0 ± 27.62 nm) and PM (892.7 ± 18.12 nm), indicating the roughness of NLC‐LA was significantly decreased (Figure 2D). All the AFM results illustrated that the surface of LA was rough with grooves, while the surface of NLC‐LA was smoother.

Additionally, AFM was employed to evaluate the interaction forces between DEX and the three carriers. Twelve particles were selected from each carrier type (LA, NLC‐LA, and PM), and the force between each carrier and DEX were measured twelve times to generate corresponding interaction force curves. The curves for NLC‐LA exhibited a smaller fluctuation range than those for unmodified LA (Figure 2E–G), and the curves for NLC‐LA stored for 6 months continued to show slight fluctuations (Figure 2H). Simultaneously, the magnitude of interaction force was calculated for each of the 12 measurements. The mean force between DEX and LA, NLC‐LA and PM were 6.35 ± 2.28 nN (RSD = 35.85%), 4.30 ± 0.96 nN (RSD = 22.30%), and 5.62 ± 2.75 nN (RSD = 49.04%), respectively (Figure 2H). The interaction force in the NLC‐LA were significantly lower than those of the LA and PM groups, with a smaller RSD (Figure 2I). Additionally, the mean force between DEX and NLC‐LA stored for 6 months was 4.19 ± 1.04 nN (RSD = 24.75%) (*p* > 0.05) in comparison to the NLC‐LA, exhibiting slight fluctuations with no significant difference (Figure 2I). The smaller particle surface area of the NLC‐LA further confirmed that NLC‐LA coated with Leu as a carrier reduced the interaction forces with DEX.

### 3.3 Coating Material Distribution of NLC‐LA

Distinct differences were observed between the Raman spectra of LA and Leu, particularly in the range of 1425–1485 cm^−1^, which corresponded to the ‐CH_2_ and ‐CH_3_ deformation vibrations of the Leu side chain. In the PM particles, correlation analysis clearly distinguished between Leu and LA particles. The green regions corresponded to LA, while the spectra extracted from the red regions matched the standard Leu spectrum, validating the discrimination capability of this method. For the NLC‐LA particles, the spectrum was predominantly similar to that of LA, and no distinct characteristic peaks of Leu were observed. The pseudo‐color distribution map, generated via correlation analysis using the Leu standard spectrum as a reference, revealed the distribution of Leu (Figure 3A–C). This suggested that Leu might be uniformly distributed across the surface of LA particles, exhibiting a distribution pattern distinct from the particle separation phenomenon observed in PM.

**FIGURE 3:**
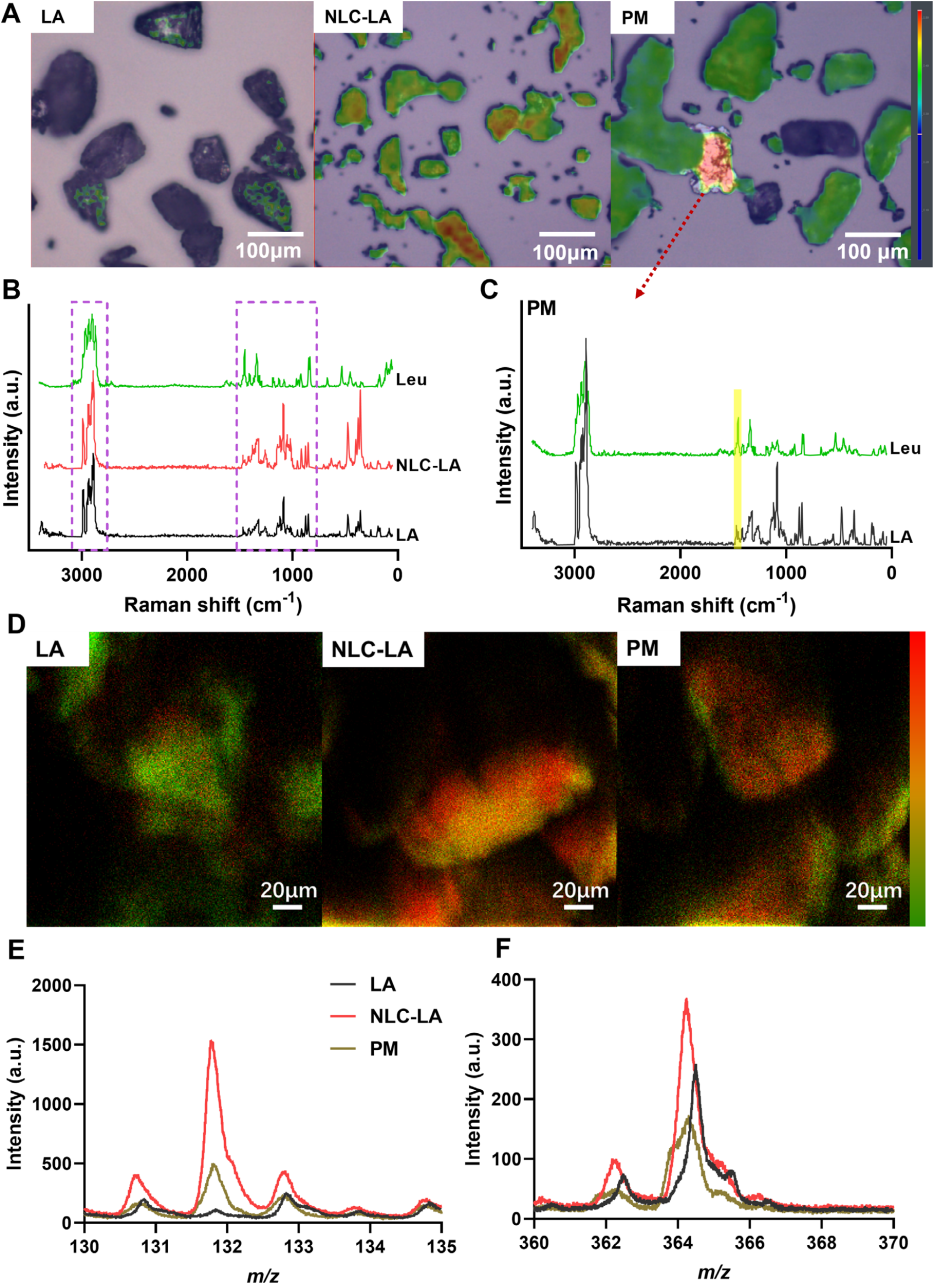
Characterization of surface material distribution of LA, NLC‐LA and PM. (A) The pseudo‐color images revealed the molecular distribution of Leu on the LA surface, which differed from the particulate separation in the solid mixture. (B, C) Comparison of the Raman spectrums of NLC‐LA and PM powders. (D) TOF‐SIMS images of three powders. (E, F) Peak intensity at 132 (*m/z*) and 365 (*m/z*) with three powders.

TOF‐SIMS was employed to investigate the surface chemical distribution of the particles. LA and Leu were identified by their characteristic sodium adduct ion at *m/z* 365 and 132, respectively. The ion intensity ratio of Leu to LA (132/365) indicated that the relative surface abundance of Leu on LA particles was significantly higher for the NLC‐LA compared with those prepared by PM (Table 1). Ion intensity mapping was further conducted to visualize the spatial distribution of the selected ions, where LA and Leu were represented by green and red signals, respectively. For the PM particles, Leu exhibited a unevenly distribution on the surface of LA particles, with distinct regions of exposed LA still observable, while no isolated LA ion signals were detected on the surface of NLC‐LA particles, suggesting that Leu might form a continuous and uniform coating layer on the LA surface (Figure 3D). The pronounced peaks at *m/z* 132 and range from 360 to 370 and 130 to 135 indicated an enriched presence of Leu in NLC‐LA, suggesting successful surface localization of Leu achieved by solvent coating rather than simple PM (Figure 3E,F and Figure S2), exhibiting significantly enhanced Leu‐related signals compared with both LA and the PM particles.

**TABLE 1:** TOF‐SIMS ion intensities of LA, NLC‐LA and PM.

Items	*m/z*	LA	NLC‐LA	PM
LA	365	257	367	171
Leu	132	247	1530	496
Intensity ratio	(132/365)	0.96	4.17	2.90

### 3.4 Crystallinity of Leu Coating Layer

SR‐µXRD was employed to investigate the local crystalline structure and phase composition of the NLC‐LA, enabling the identification of crystallinity, polymorphic forms, and spatial heterogeneity between the surface coating and the underlying matrix. Results showed that compared to LA, PM particles exhibited distinct Leu characteristic peaks at 6.68° and 11.34°, displaying a pattern nearly identical to Leu. However, no characteristic peaks of Leu and Leu‐H were detected in the NLC‐LA particles, which exhibited a pattern identical to LA with consistent characteristic peaks. This might result from excessive LA response masking the Leu peaks (Figure 4A). In addition, a 2D scan of the NLC‐LA powder was performed, selecting specific granular regions to obtain a total 36 diffraction patterns (Figure 4B), revealing characteristic peaks for Leu‐H that were found in diffraction patterns (numbers 17–21 and 27–32). The diffraction pattern 30 was selected from these to highlight the characteristic peaks of NLC‐LA, demonstrating that the crystals of Leu‐H appeared with the characteristic peak at 9.41° (Figure 4C–E). This revealed the presence of partial Leu‐H crystallization on the surface of the coated LA. Analysis of SR‐µXRD measurements for NLC‐LA and PM powder revealed that PM exhibited distinct Leu characteristic peaks in comparison to LA, suggesting the presence of significant Leu particles. Although Leu‐H characteristic peaks were detected on NLC‐LA, their intensity was low and the detection area was small, highlighting a reduction in the diffraction lattice volume of Leu. In summary, the characteristic sharp diffraction peaks of Leu were still retained in PM particles, but these peaks were significantly weakened or disappeared in NLC‐LA particles, an aspect that was particularly evident after 2D scanning and regional integration. The 2D diffraction patterns exhibited diffuse scattering characteristics, demonstrating that Leu in NLC‐LA primarily existed in a highly dispersed, low‐crystallinity, or amorphous state on the LA particles surface rather than forming a crystalline coating.

**FIGURE 4:**
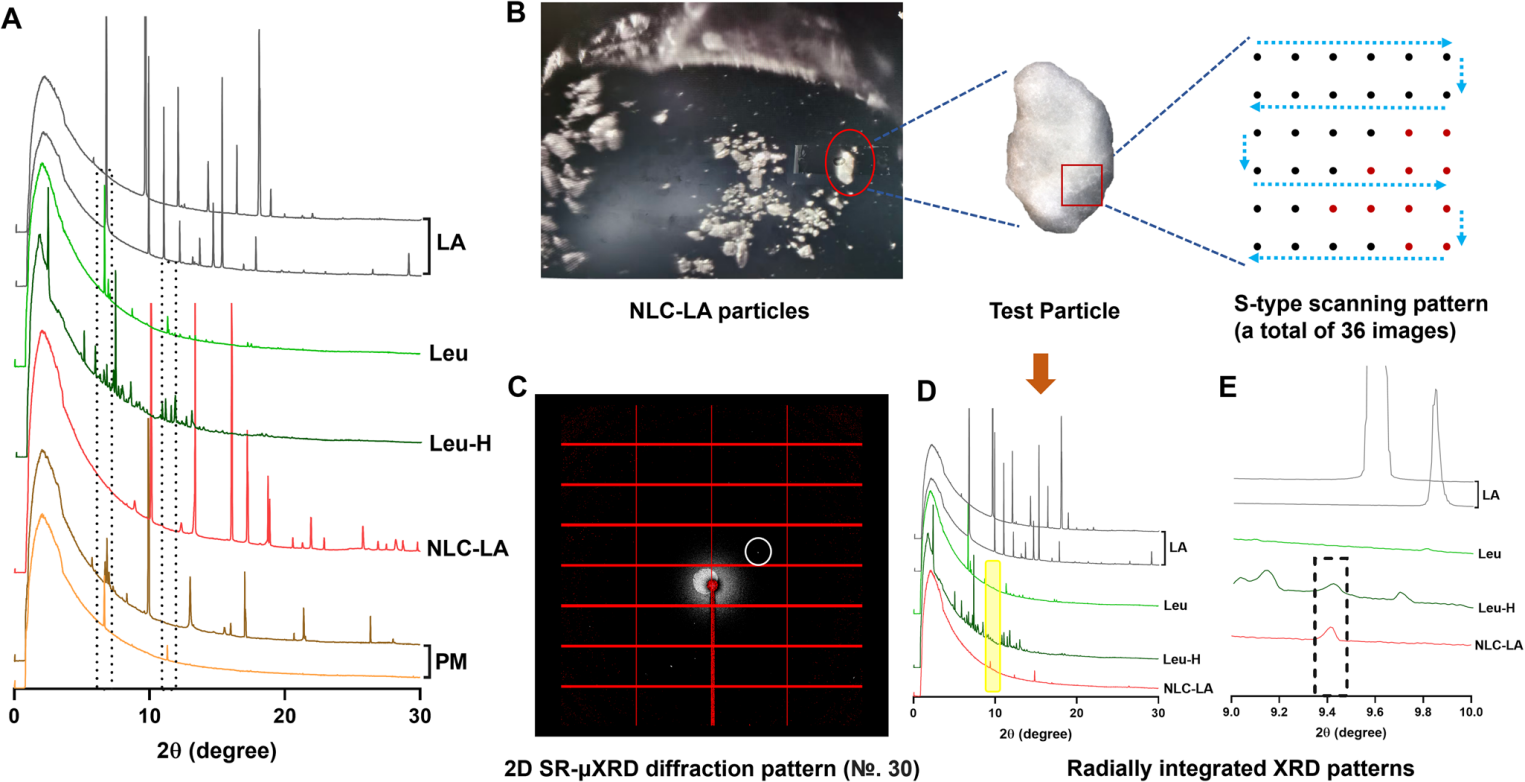
Characterization of LA, NLC‐LA and PM particles in SR‐µXRD. (A) XRD patterns of LA, Leu, Leu‐H, NLC‐LA and PM powders under single‐point scanning mode. (B) Under 2D scanning mode, S‐shaped scanning pattern to obtain diffraction patterns of NLC‐LA. (C) 2D diffraction patterns of NLC‐LA at figure 30 as typical diagram. (D) Comparison of radially integrated XRD patterns of LA, Leu, Leu‐H, and NLC‐LA powders and (E) magnified image from 9° to 10°.

### 3.5 NLC‐LA Coating Thickness Using SR‐µCT and FIB‐SEM

SR‐µCT‐based quantification confirmed treatment‐induced micro‐morphological evolution of LA particles. Both S‐LA and NLC‐LA exhibited overall particle shrinkage, while NLC‐LA showed a stronger tendency toward surface‐texture loosening as reflected by a decrease in 3D solidity. Detailed SR‐µCT statistics were provided in the “*Statistical analysis of particle morphological parameter evolution*” of Supplementary Material. To investigate the underlying mechanisms, the correlation between initial morphological parameters and their subsequent variations was analyzed, revealing a convergent “chemical polishing” mechanism. Regardless of the solvent used, particles with an initial sphericity below 0.80 exhibited a positive increase in this parameter, whereas those with higher initial sphericity underwent minor negative changes (Figure 5A). This trend indicated the preferential removal of high‐energy sites such as edges and corners from irregular particles during the treatment process. Threshold‐based statistics using a discrimination value of 1 × 10^−6^ further differentiated the treatment effects. Macroscopically, skeletal integrity was preserved in both groups, where over 97% of particles showed no significant change, while the groups diverged significantly in 3D solidity from a microscopical viewpoint (Figure 5B and Figure S3A). The S‐LA group favored densification with a 35.1% positive change, suggesting the filling of surface defects. Conversely, the NLC‐LA group was dominated by loosening or etching as evidenced by a 48.0% negative change, confirming that NLC‐LA induced mesoscopic porosity alongside surface polishing.

**FIGURE 5:**
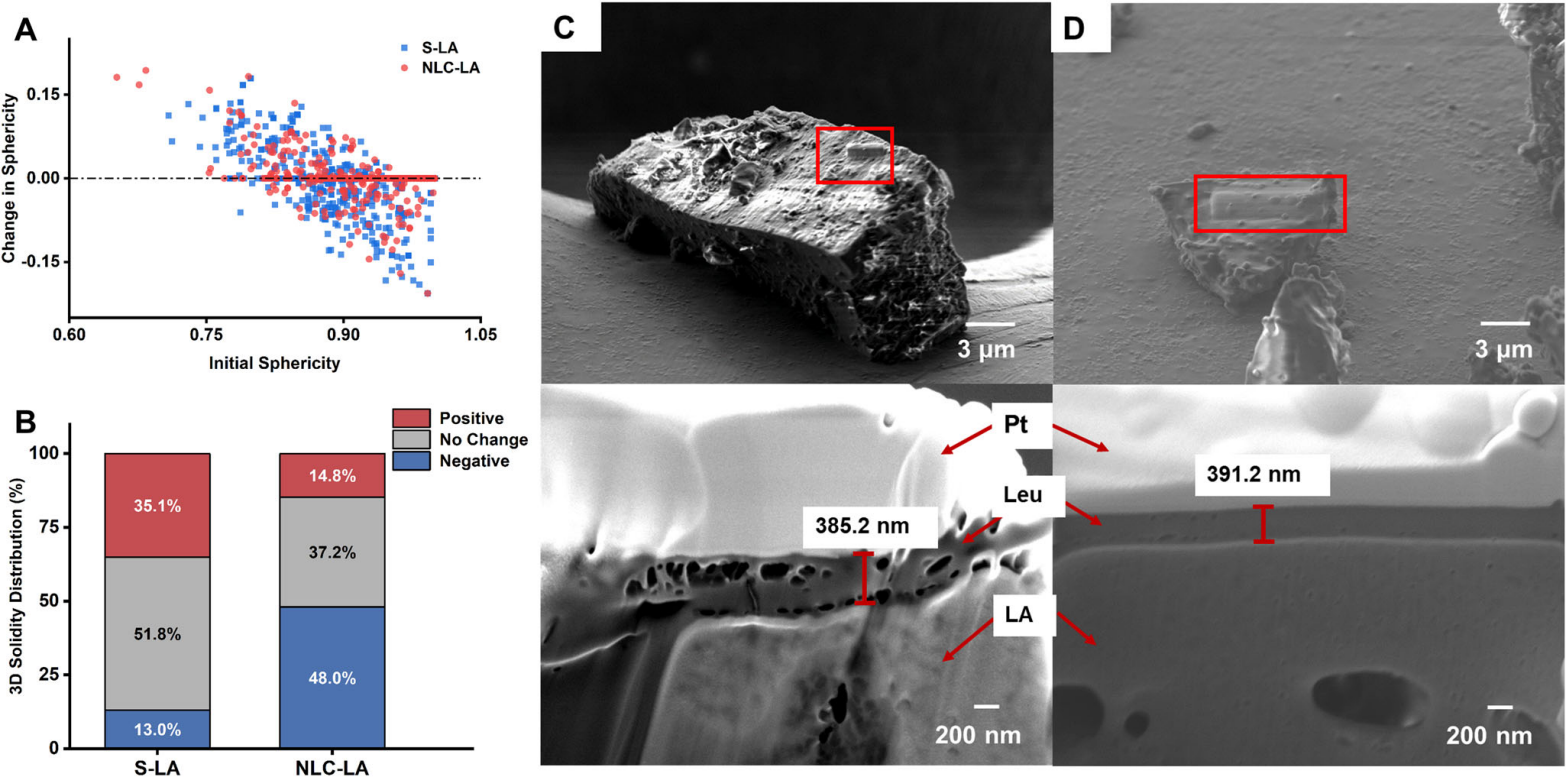
Analysis and verification of NLC‐LA particle coating thickness based on SR‐µCT and FIB‐SEM. (A) Initial‐value dependent convergence of sphericity. (B) Categorical statistics of changes in 3D solidity, classified by a threshold of 1 × 10^−6^. (C, D) SEM image of the cutting positions for two NLC‐LA particles and in situ cross‐section distribution map of the corresponding particles.

Based on SR‐µCT results, the total LA surface area (A_total_) and volume (V_total_) of a single CT were determined to be 9.12 × 10^8^ µm^2^ and 1.85 × 10^10^ µm^3^, comprising 20,492 LA particles. Additionally, the densities of LA and Leu were 1.768 g/cm^3^ and 1.293 g/cm^3^, respectively. Calculations from the method yielded a coating thickness of 27.7 nm for NLC‐LA particles. Based on the molecular size of Leu‐H, the membrane layer range was determined to be 18–54 layers.

Systematic analysis of FIB‐SEM cross‐section results revealed that after removing the outermost protective Pt deposition layer (appearing as bright white regions), a continuous, elongated layered structure with distinct gray‐scale differences distributed along the particle surface emerged in the cross‐section of the NLC‐LA sample. This layer exhibited significant contrast and morphological differences from the LA, indicating a composition distinct from the LA matrix and suggesting it might represent a heterogeneous phase embedded within the matrix surface. Considering the NLC‐LA particles preparation process and characteristics of the Leu solvent coating technique, it was reasonable to infer that this elongated layer corresponds to the Leu coating on the particle surface. Further quantitative measurement of this layer revealed that the Leu coating exhibited a relatively continuous and uniform distribution on the particle surface, with an average thickness of approximately 380 nm (Figure 5C,D). This result demonstrated that the solvent coating method could successfully construct a nanoscale Leu‐coated layer on the surface of LA particles, providing a structural foundation for subsequent regulation of the surface properties of LA particles.

Combining CT calculation results revealed significant discrepancies with FIB‐SEM test outcomes. Analysis suggested this might stem from the coating thickness measured via CT (approximately 27.7 nm) potentially representing an average shell thickness constrained by material contrast and segmentation accuracy limitations. In contrast, the FIB‐SEM cross‐section captures locally visible coating‐related layers (approximately 380 nm), which might incorporate surface micro‐protrusions and milling‐induced artifacts (e.g., redeposition and interface mixing), which could enlarge the apparent thickness of the coating region. The difference between the two measurements does not necessarily indicate an inconsistency, but rather reflects the distinction between an averaged equivalent thickness obtained from a large particle population and a local cross‐sectional thickness obtained from a single particle region. Nevertheless, both results support the presence of a nanoscale leucine coating on lactose particles.

### 3.6 Pharmacokinetics of DEX‐NLC‐LA in Rats

The pH of DEX DPI was measured to be 5.76, which fell within the acceptable range for inhalation preparations (about 4.0‐8.0) [36], confirming that it may not cause respiratory irritation and lungs. Pharmacokinetic studies were conducted to evaluate the absorption and metabolism between DEX‐LA and DEX‐NLC‐LA in rats. Pharmacokinetic curves for both groups demonstrated biphasic release characteristics (Figure 6A,B). Analysis of individual rat absorption curves revealed that this biphasic pattern was more pronounced in the DEX‐LA group (Figure S4). The area under the concentration‐time curve (AUC) was higher for the DEX‐NLC‐LA group (613.6 ± 231.7 ng/mL·h) than for the DEX‐LA group (442.9 ± 98.84 ng/mL·h), representing an approximately 1.5‐fold increase (Figure 6C and Table S4). Additionally, the peak plasma concentration (C_max_) values for the inhaled DEX‐LA and DEX‐NLC‐LA groups were 131.6 ± 60.16 ng/mL and 137.3 ± 45.56 ng/mL, respectively (Figure S5A and Table S4). The time to reach peak drug concentration (T_max_) between the two groups was 0.75 and 0.19 h, indicating more rapid absorption via inhaling DEX‐NLC‐LA (Figure S5B and Table S4). However, no significant differences were observed in half‐life (T_1/2_) and mean residence time (MRT) between the two groups (Figure S5C and Table S4). Collectively, these results indicated that inhaled DEX‐NLC‐LA enhanced the plasma exposure of DEX. This finding correlates with the in vitro aerodynamic deposition analysis, where DEX‐NLC‐LA showed a higher FPF of 50.15% compared to 23.23% for DEX‐LA. The positive trend between the higher FPF and the increased AUC suggests that improved aerosol performance led to greater pulmonary deposition and, consequently, higher plasma drug exposure (Figure 6D). Therefore, the DEX‐NLC‐LA was delivered more efficiently to the lungs, facilitating enhanced absorption into the systemic circulation.

**FIGURE 6:**
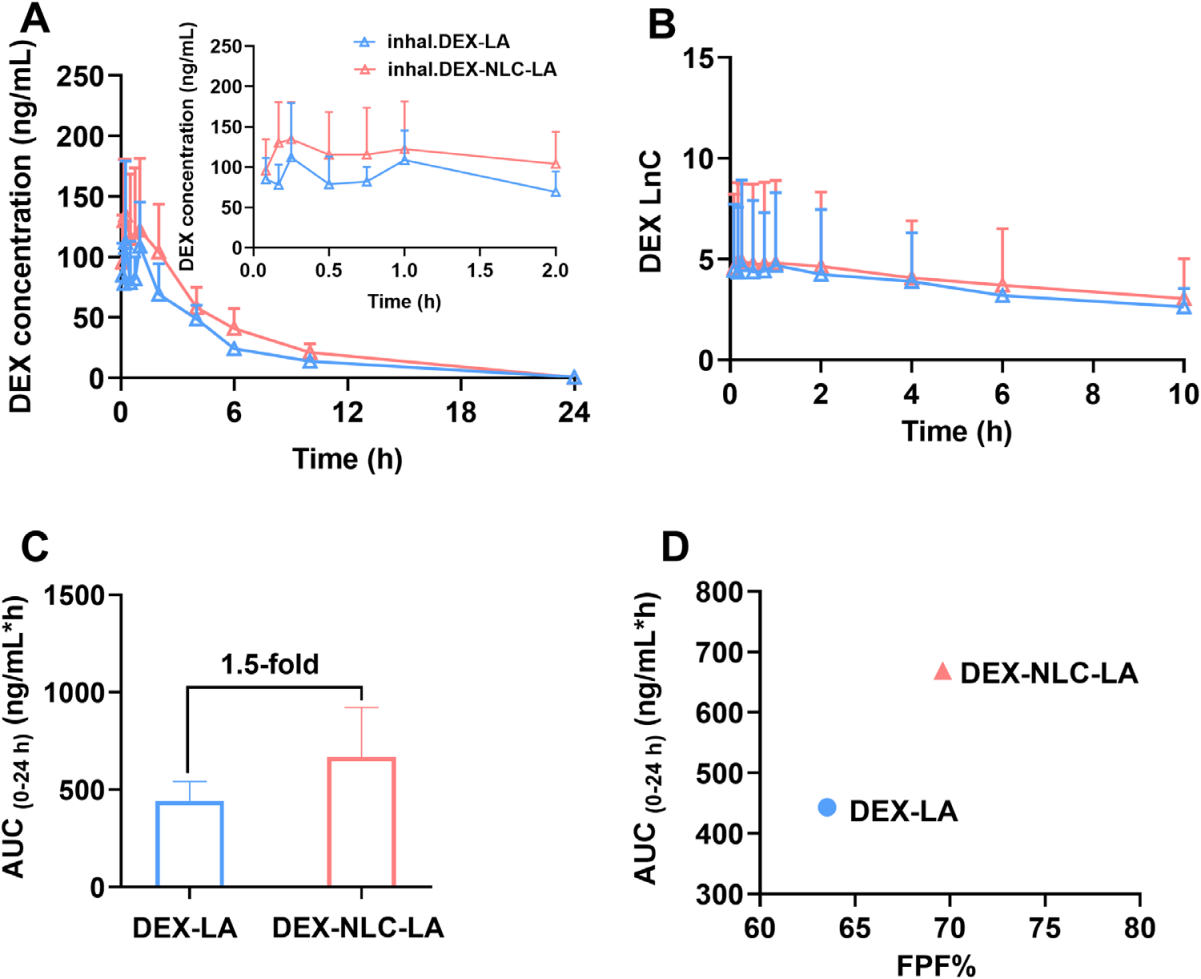
Pharmacokinetics evaluation of DEX‐NLC‐LA in rats (*n* = 3). (A, B) Plasma concentration‐time profiles and LnC‐time profiles of inhal. DEX‐LA and inhal. DEX‐NLC‐LA groups. (C) Comparison of AUC parameters between inhal. DEX‐LA and inhal. DEX‐NLC‐LA groups. (D) Scatter plot showing the trend between FPF in vitro and AUC in vivo for DEX‐LA and DEX‐NLC‐LA formulations.

### 3.7 Efficacy of DEX‐NLC‐LA

The efficacy of DEX‐NLC‐LA (0.1 and 0.3 mg/kg) in preventing and treating HALI model was evaluated via inhalation. The blank group exhibited normal lung tissue appearance with intact alveolar structures, thin septa, and minimal inflammatory cell infiltration, while the model group showed marked congestion and dark red discoloration of lung tissue. H&E sections revealed disrupted alveolar structures, thickened septa, and pronounced inflammatory reactions, indicating successful establishment of HALI model. Compared to the model group, the intravenous DEX group (3 mg/kg) showed reduced pathological damage in lung tissue, though some structural disorganization and inflammatory changes remained visible. Both inhalation treatment groups (0.1 and 0.3 mg/kg) demonstrated more pronounced protective effects, in which the lung tissue appearance and histological structure approached normal levels, with significantly reduced inflammation and alveolar destruction (Figure 7A).

**FIGURE 7:**
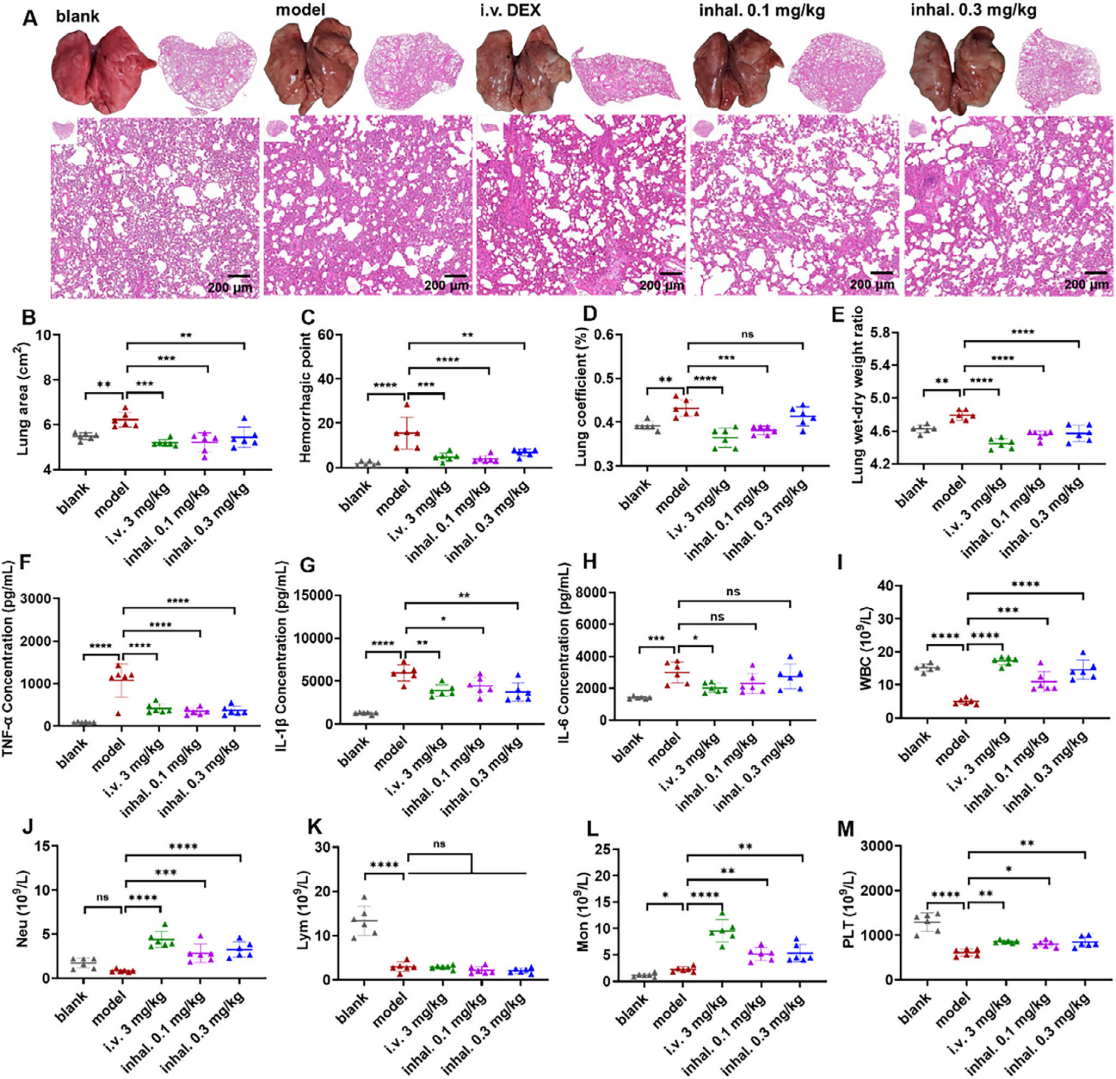
Therapeutic effect of DEX‐NLC‐LA to alleviate HALI in rats. (A) Typical appearance and H&E images of lungs from rats in blank, HALI model, i.v. DEX, and inhal. DEX‐NLC‐LA (0.1 and 0.3 mg/kg) groups (scale bar, 200 µm). (B, C) Comparison of lung appearance score in five groups, including hemorrhagic point and lung area. (D, E) Expression of lung coefficient and lung wet‐dry weight ratio of rats. (F‐H) Inflammatory factors level (TNF‐α, IL‐1β and IL‐6) in rat lung. (I‐M) Blood indicators in rat serum, including WBC, Neu, Lym, Mon, and PLT. (*n* = 6, *ns* = no significant, ^*^ *p* < 0.05, ^**^ *p* < 0.01, ^***^ *p* < 0.001, ^****^ *p* < 0.0001).

Lung area and hemorrhagic points were compared across groups to assess the efficacy of DEX‐NLC‐LA. The model group exhibited significantly larger lung area (6.22 ± 0.33 cm^2^) compared to the blank group (4.99 ± 0.15 cm^2^). Additionally, the number of hemorrhagic points (15.58 ± 7.12) was markedly higher than in the blank group (1.83 ± 0.98) (*p* < 0.0001), confirming the successful establishment of the HALI model. Both the positive control group (i.v. DEX) and the inhalation groups (inhal. 0.1 and 0.3 mg/kg) exhibited significantly reduced lung area and fewer hemorrhagic points compared to the model group, indicating that both intravenous injection and inhaled DEX‐NLC‐LA effectively ameliorate HALI (Figure 7B,C). Further measurement of pulmonary edema parameters revealed that the model group had a significantly higher lung wet‐dry weight ratio (4.79 ± 0.06 vs. 4.63 ± 0.05) and lung coefficient (0.43 ± 0.02% vs. 0.39 ± 0.01%) than the control group (*p* < 0.01). Inhaled DEX‐NLC‐LA (0.1 and 0.3 mg/kg) significantly reduced both the lung wet‐dry weight ratio and lung coefficient compared to the model group (*p* < 0.001) (Figure 7D,E). These data confirmed that inhaled DEX‐NLC‐LA effectively alleviated pulmonary edema.

Pulmonary inflammatory factor levels were also significantly elevated in the model group, with higher TNF‐α, IL‐1β, and IL‐6 concentrations compared to the control (*p* < 0.001). Inhaled DEX‐NLC‐LA significantly reduced the levels of all three cytokines relative to the model group, demonstrating its anti‐inflammatory efficacy in HALI (Figure 7F–H). Focusing on the effects of the treatments on blood parameters in model rats, the results showed that inhaled DEX‐NLC‐LA restored white blood cell, neutrophil, lymphocyte, monocyte, and platelet counts toward normal levels compared to the model group, indicating that DEX effectively alleviated inflammation levels and restored the body's immune balance (Figure 7I–M). Notably, compared with the high‐dose intravenous positive control (3 mg/kg), inhaled DEX‐NLC‐LA at 1/30 of the intravenous dose (0.1 mg/kg) effectively alleviated pulmonary edema and inflammation in the HALI model.

## 4 Discussion

LA is the most commonly used excipient in carrier‐based DPI formulations [37]. In contrast to conventional blending, where strong drug‐carrier adhesion in DEX‐PM resulted in predominant deposition in the throat and pre‐separator (67.43%), surface modification of LA significantly improved aerosolization performance. Among the four modification strategies evaluated, Leu hydrochloride ethanol solution treatment was the most effective in enhancing the aerodynamic performance of DEX while maintaining good powder flowability. Previous studies had shown that MgSt (0.25%, w/w) coating applied through multiple (ten) successive cycles improved the aerodynamic performance of beclomethasone dipropionate DPIs and reduced LA surface roughness, while this approach was limited by the need for repeated coating cycles during LA processing [38, 39]. Interestingly, in this study, LA was treated only once using a Leu hydrochloride ethanol solution, with a low Leu‐H content of 0.05% (w/w, 50 µL, 1 g LA), to prepare DEX‐NLC‐LA, which exhibited favorable aerodynamic performance (FPF = 50.15%). This favorable result was attributed to the successful surface modification of the LA carrier. As a surface modifier, Leu effectively reduced the surface energy of LA particles and masked high‐energy adhesion sites [40, 41], thereby weakening the interfacial interactions between DEX and LA. Consequently, DEX detachment during aerosolization was facilitated, leading to a significantly marked improvement in FPF.

Compared with LA, NLC‐LA particles exhibited markedly reduced surface grooves on partial particles, which was attributed to the coating process in the Leu solution, during which heating and stirring caused solvent evaporation, leading to continuous accumulation and backfilling of Leu crystals onto the LA surface. In addition, the interaction force between NLC‐LA and DEX was significantly reduced, indicating that the NLC method effectively smoothed the LA surface by masking surface grooves and high‐energy defect sites [42]. The smooth surface not only reduced mechanical interlocking between particles but also decreased contact area [8, 24], thereby improving powder flowability and atomization performance. SR‐µCT analysis of a large population of LA, S‐LA, and NLC‐LA particles revealed the coating process did not induce pronounced changes in the global particle structure. However, the three‐dimensional compactness predominantly exhibited negative shifts, suggesting that the Leu‐H ethanol coating induced surface loosening of LA particles rather than dense encapsulation. Additionally, TOF‐SIMS confirmed a uniform distribution of Leu on the NLC‐LA particles surface and the homogeneity of the coating process. Furthermore, SR‐µXRD analysis revealed characteristic diffraction peaks corresponding to Leu‐H on the surface of NLC‐LA particles, confirming the formation of a stable and homogeneous surface modification layer. Collectively, these findings indicated that the Leu‐H layer preferentially deposited at high‐energy sites on the LA surface for NLC‐LA particles, thereby reducing drug‐carrier cohesion (and adhesion) [5, 24] and decreasing the effective contact area between DEX and LA. This weakened interfacial interaction facilitated drug detachment during aerosolization, resulting in a pronounced enhancement of aerosol performance, as reflected by a significant increase in FPF from 23% to 50%. Interestingly, the combined evaluation using SR‐µCT and FIB‐SEM determined the coating thickness of Leu on the surface of NLC‐LA particles, confirming a nanoscale coating.

Although this process had achieved nanometer‐scale coating using a low Leu content, further considerations are required for potential clinical application. In particular, the critical parameters of manufacturing process were elaborated, including controlling the FPF at 40%–50%, the CI of flowability at 20%–25%, the RSD of content uniformity below 2%, and residual solvent below 2%. And multi‐cycle particle smoothing via high‐shear mixing was effective; however, our single‐step Leu deposition from ethanolic solution offered comparable surface modification with simplified operation, reduced processing time, and lower energy consumption. Rapid ethanol evaporation further minimizes moisture‐related risks such as LA recrystallization and uncontrolled aggregation. While industrial‐scale cost‐benefit analysis must still account for solvent recovery, handling requirements, and scale‐up feasibility, this single‐step strategy represents a potentially more efficient route for tailoring lactose carrier surfaces.

Furthermore, pulmonary delivery of DEX‐NLC‐LA demonstrated enhanced bioavailability, attributed to differences in in vitro aerodynamic behavior. The magnitude of in vitro FPF values reflected particle deposition patterns, with a higher FPF (50.15%) reflecting greater penetration of DEX‐NLC‐LA particles into the terminal bronchioles and alveolar regions [43, 44]. Upon pulmonary delivery, particles evade mucociliary clearance and directly cross the alveolar epithelium into the circulation. This is facilitated by the vast surface area and rich blood supply of the deep lung, thereby avoiding first‐pass metabolism [45, 46, 47]. Particles with poor in vitro deposition (DEX‐LA) were more likely to deposit in the upper airway and subsequently swallowed into the gastrointestinal tract, leading to first‐pass effects and reduced bioavailability, and a biphasic absorption characteristic. Based on this finding, during efficacy evaluation, pulmonary delivery of a low dose (0.1 mg/kg) DEX‐NLC‐LA efficiently alleviated HALI. Compared to intravenous injection (positive control, 3 mg/kg), DEX administrated by inhalation at 1/30 of the intravenous dose still effectively decreased pulmonary edema and inflammatory cytokine levels in HALI rats. The tissue distribution results indicated that approximately 89% of the DEX DPI reached the pulmonary region [34], achieving high therapeutic efficacy. Therefore, the reduction of the dose to 1/30 of the intravenous equivalent is not merely nominal, but rather a conclusion supported by the integrated evidence of pharmacological efficacy, pharmacokinetics, and tissue distribution. As a glucocorticoid drug, DEX requires precise dosing to maximize efficacy while minimizing systemic toxicity [34, 48]. In addition, the assessment of the pulmonary tolerability of the Leu‐H coated carrier and DEX DPI should be explored in subsequent studies. The NLC strategy developed in this study addresses this need by simplifying manufacturing and enabling stable DEX formulations. These novel formulations facilitated efficient pulmonary delivery and effectively alleviated HALI at significantly reduced doses.

## 5 Conclusion

In conclusion, this study demonstrated that NLC strategy effectively resolves the long‐standing trade‐off between high pulmonary deposition and adequate powder flowability in DPIs. A continuous and uniform Leu nanolayer was successfully formed on LA particles using a minimal amount of Leu. This resulted in DEX‐NLC‐LA formulation, which increased the FPF from approximately 23% to 50%, thereby achieving simultaneous improvements in aerosolization efficiency. Mechanistic investigations using complementary structural techniques confirmed that NLC‐LA did not alter particle morphology but instead reduced surface roughness and interparticle adhesion through the formation of an ultrathin Leu coating, thus facilitating drug detachment during aerosolization. Importantly, these physicochemical advantages translated into superior in vivo outcomes, yielding a 1.5‐fold increase in DEX bioavailability and reducing the therapeutic dose to 1/30, all while maintaining efficacy in a HALI model. Overall, this work establishes NLC‐LA as a rational and scalable surface‐engineering platform for developing high‐performance DPI formulations, particularly for cohesive or poorly flowable drugs.

## Author Contributions

**Q.N**.: conceptualization, methodology, software, data curation, visualization, Writing – review and editing. **J.L**.: writing, investigation, software, data curation, writing – review and editing. **X.Z**. and **S.Y**.: data curation, investigation, writing – review and editing. **H.S**., **B.J**., **X.C**., **X.R**. and **L.S**.: data curation, writing – review and editing. **L.W**., **C.W**. and **J.Z**.: conceptualization, methodology, revising‐ review and editing.

## Conflicts of Interest

The authors declare no conflicts of interest.

## Supporting information




**Supporting File**: advs76929‐sup‐0001‐SuppMat.docx.

## Data Availability

The data that support the findings of this study are available on request from the corresponding author. The data are not publicly available due to privacy or ethical restrictions.
