# Supplementary material for: Nanometer Layer Coating to Lactose Particles for Optimization of Dexamethasone Pulmonary Delivery
Source: Adv Sci (Weinh). 2026 Aug 3;13(60):e76929. doi: 10.1002/advs.76929 (PMC13430934; doi:10.1002/advs.76929)
Supplement: Supplementary file 1 [file ADVS-13-e76929-s001.docx]

**Supplementary Material**

***Determination of residual solvent and degradation formation.*** The content of the degradation formation 5-HMF was determined by UV spectrophotometry (Hitachi Ltd., UH-5300, Japan). The calibration curve was established at concentrations of 0.5, 1, 2, 4, 6, 8, and 10 μg/mL, with the detection wavelength set at 292 nm. The analytical method was validated over a linear concentration range of 0.5-10 μg/mL (*r* = 0.9984). Additionally, the pH of DEX DPI was tested using professional pH meter (pp-20, Sartorius, Shanghai, China) (*n* = 3).

The residual ethanol content in the samples was determined using headspace gas chromatography coupled with flame ionization detection. Chromatographic separation was performed on an Agilent DB-624 capillary column (30.0 m × 0.53 mm i.d., 3.00 μm film thickness). High-purity nitrogen was used as the carrier gas at a constant flow rate of 2.8 mL/min. Headspace samples were introduced into the GC system in split mode at a split ratio of 5: 1, and the GC inlet temperature was set at 250°C. The oven temperature was initially maintained at 40°C for 6 min, increased to 220°C at a rate of 20°C/min, and then held at 220°C for 10 min. Detection was performed using a flame ionization detector maintained at 250°C. For headspace sampling, the sample vials were equilibrated at 80°C for 10 min. The sample-loop and transfer-line temperatures were maintained at 110°C and 120°C, respectively, and a 1.0 mL sample loop was used. The calibration curve was established at concentrations of 0.2, 0.4, 0.6, 0.8, and 1.0 μg/mL, and the resulting solution (5.0 mL) was accurately transferred into a 20 mL headspace vial, which was immediately sealed for analysis.

***NGI tests.*** The aerodynamic behaviors of all DEX DPIs were tested using the Next Generation Impactor (NGI) (Copley Science Ltd., Nottingham, UK). A flow rate was set to 60 L/min using a vacuum pump and the inhalation process lasted for 4.0 s with a TPK critical flow controller. Capsules (#3 HPMC, Empty Vcaps^®^ Plus Capsules, Jiangsu, China) were loaded with 10 mg powder. Five capsules were inhaled per experiment using a Breezhaler® device (*n* = 1). After the test, all components of the NGI were rinsed with the mixed solvent (50% ethanol), and transferred to a volumetric flask and diluted to the marked volume. The solution was centrifuged at 12,000 rpm for 10 min, and the supernatant was analyzed by HPLC (Agilent Technologies Co. Ltd., Palo Alto, USA) to determine the drug concentration. The concentrations of DEX standard solution were 0.5, 1, 2, 5, 10, 20, 50, and 100 μg/mL and determined by HPLC using a Kinetex XB C₁₈ column (50 × 4.6 mm, 5 μm) maintained at 40°C, with a mobile phase consisting of water and acetonitrile at a volume ratio of 70: 30 at a flow rate of 0.8 mL/min. The wavelength of 240 nm was performed using a UV detector, and the injection volume was fixed at 10 μL.

***Carr's Index.*** A 5 mL graduated cylinder was accurately weighed and recorded as m_0_. The powder sample to be tested was slowly and gently added into the graduated cylinder, and the initial volume of the powder (V_0_) was recorded before weighing the total mass as m_1_. The graduated cylinder was then tapped vertically until no significant change in volume was observed, with the final volume (V_1_) noted. The mass of the powder was determined as the difference between the mass of the graduated cylinder before and after powder addition. Carr's Index (CI) of all powders were further calculated using the formulas (1) ~ (3):

Bulk density (ρ_ᵦ_): ρ_ᵦ_ = m_0_ / V_0_………………………………………........................(1)

Tap density (ρ_ₜₐₚ_): ρ_ₜₐₚ_ = m_1_ / V_1_………………………………………........................(2)

Carr's Index (CI): CI = (ρ_ₜₐₚ_ - ρ_ᵦ_) / ρ_ₜₐₚ_ × 100%.............................................................(3)

***SM-CT1. Materials and sample preparation.*** For sample preparation, two portions of SV010 lactose powder (50 mg each) were accurately weighed and placed into separate capsule shells. Following the completion of the initial SR-μCT imaging, 200 μL of ethanol was added to the first portion to obtain the S-LA sample, while 200 μL of leucine hydrochloride ethanol solution was added to the second portion to produce the NLC-LA sample. The mixtures were agitated every 20 min to ensure homogeneous dispersion. Subsequently, the samples were dried in an oven at 50°C for 2 h to obtain particles for further SR-μCT characterization.

***SM-CT2. Detailed SR-μCT acquisition parameters.*** SR-μCT was conducted at the BL13HB beamline of the Shanghai Synchrotron Radiation Facility, abbreviated as SSRF. The following datasets were acquired and analyzed, including LA, S-LA, and NLC-LA. The photon energy was 22 keV. For each scan, 720 projections were acquired using a CCD camera with a resolution of 2048 by 2048 pixels. The camera model was pco.2000 and it was manufactured by PCO AG in Kelheim, Germany. The effective pixel size was 3.25 μm. The exposure time was 100 ms, and the sample-to-detector distance was 11 cm. Flat-field images and dark-field images were collected to correct electronic noise and variations in X-ray source brightness.

***SM-CT3. Reconstruction and preprocessing workflow***. Propagation-based phase-contrast extraction and slice reconstruction were performed using PITRE version 3.1 developed at SSRF. Projection images were converted into sinograms, and reconstruction was carried out using filtered back-projection. The rotational center axis was continuously adjusted to ensure that the reconstructed slice structures were complete. Reconstructed three-dimensional images were cropped and grayscale-corrected to minimize background while retaining the full particle structure in each cropped slice. Grayscale values were normalized to the range from 0 to 255 by converting the data type from 32-bit to 8-bit. All reconstructed slices were imported into Avizo software provided by Thermo Fisher Scientific for three-dimensional rendering, visualization, and quantitative analysis.

***SM-CT4. Single-particle matching and statistical analysis details***. Raw morphological parameters were extracted as described in Section 2.2.3. To mitigate edge effects and outlier interference, particles were filtered to retain only those within the 20th to 50th percentile of the volume distribution. A multidimensional feature space was constructed, and geometrically stable parameters such as volume and equivalent diameter were used as matching features. One-to-one particle mapping between pre-treatment and post-treatment datasets was achieved via Z-score normalization and Euclidean distance minimization. Non-increasing physical constraints were imposed on volume and dimensional metrics to eliminate erroneous matches. To address zero-inflation in parameter variations, an epsilon threshold equal to 1×10 to the power of minus 6 was applied to isolate effectively responsive particles. Gaussian kernel density estimation was used with independent normalization to characterize variation distributions, followed by initial size-dependency analysis and threshold-based classification of change types, including positive, negative, and negligible changes.

***Calculation of the coating thickness of NLC-LA particles.*** The total surface area (A_total_) and total volume (V_total_) of LA in a single CT scan were obtained. Based on the densities of LA (ρ_LA_) and Leu (ρ_Leu_), the Leu coating thickness in the NLC-LA sample was calculated via equal (4) ~ (10).

The quality of a single CT scan (M) was calculated via equal (4):

$M= V\text{total}\times\text{ρ}\text{LA}$……….....……...……………………………........................(4)

For the NLC-LA sample, Leu-H was coated onto the surface of LA (0.1: 1 g, w/w). The surface area of the LA (0.999 g) was denoted as A.

$A= A\text{total}\times\frac{0.999}{M}$………………...……………………………........................(5)

The volume of Leu (V_Leu_) was calculated as follow:

$V\text{Leu}= \frac{m\text{Leu}}{\text{ρ}\text{Leu}}$………..........………...……………………………........................(6)

Coating thickness (t) was calculated as:

$t= \frac{V\text{Leu}}{A}$………………...................……………………………........................(7)

In the CCDC database, the size of Leu-H was found, as follows: a=11.152 Å, b=5.116 Å, c=15.405 Å (10 Å =1 nm) (α = 90°, β = 90°, γ = 90°)

Nanometer layer coating thickness (n) for NLC-LA particle was calculated as:

$n\text{a}= \frac{t}{a}$………………...………...…………………………….........................(8)

$n\text{b}= \frac{t}{b}$………………...…………....…………………………........................(9)

$n\text{c}= \frac{t}{c}$…………...……………...……………………………........................(10)

***FIB-SEM.*** In a focused ion beam–scanning electron microscopy (FIB-SEM) dual-beam system, the term “dual-beam” refers to the integration of a focused ion beam and an SEM electron beam, whose coupled operation constitutes the dual-beam configuration ^[1]^. In such a system, the electron beam is mounted vertically, while the ion beam is oriented at a fixed angle relative to the electron beam. By adjusting the specimen stage tilt, specimen height, and fine beam alignment, the region of interest can be positioned at the coincident focal point of both beams, enabling simultaneous SEM imaging and FIB milling ^[2]^. The micro- and nanofabrication capability of the system primarily relies on the cascade collision phenomenon induced by the focused ion beam ^[3]^. The operating principle of the ion beam is analogous to that of the electron beam, in that both functions are realized through the interaction between a focused beam spot and the specimen surface. During cascade collisions, displaced atoms traveling toward the specimen surface may pass through lattice channels and escape from the surface as sputtered atoms or secondary ions, thereby enabling material removal and micro/nanoscale machining ^[4]^.

***Concentration determination*** ***of plasma.*** The LC-MS/MS (Agilent Technologies Co. Ltd., Palo Alto, USA) analysis was employed to determine DEX in rat plasma, monitoring DEX (*m/z* 393→147.1) and internal standard (*m/z* 361.1→147). Briefly, plasma sample (20 μL) was mixed with diluent (10 μL), internal standard solution (prednisolone, 1250 ng/mL, 10 μL), and acetonitrile (60 μL). The mixture was vortexed for 120 s, then centrifuged at 12,000 rpm at 4°C for 15 minutes. The supernatant was collected for analysis. Chromatographic separation was performed using a Kinetex-XB C₁₈ column (100 × 4.6 mm, 5 μm) maintained at 40°C with 0.8 mL/min and the mobile phase consisted of 0.1% formic acid and acetonitrile (60: 40, v/v) with an injection volume of 10 μL.

**Table S1**	The flowability and aerodynamic parameters of powders.

| Coating method | Surface modifiers | DPI | CI (%) | Emptying rate (%) | Percentage content of throat and prs-s (%) | FPF (%) |
| --- | --- | --- | --- | --- | --- | --- |
| - | - | DEX | 45.87 ± 0.23 | 90.00 ± 1.81 | 5.40 | 68.87 |
|  | - | DEX-LA | 18.00 ± 1.00 | 99.00 ± 0.66 | 67.43 | 20.23 |
| Solid | SA | DEX-LC-LA | 27.31 ± 2.84 | 99.90 ± 0.14 | 72.56 | 16.92 |
|  | PA |  | 29.03 ± 3.48 | 99.86 ± 0.20 | 80.63 | 14.25 |
|  | SAc |  | 30.23 ± 2.45 | 100.1 ± 0.17 | 70.54 | 19.13 |
| Suspension | solvent |  | 24.23 ± 1.58 | 99.01 ± 0.23 | 65.27 | 18.82 |
|  | Leu |  | 26.32 ± 4.45 | 99.72 ± 0.11 | 58.33 | 32.56 |
|  | MgSt |  | 24.23 ± 4.58 | 99.68 ± 0.40 | 61.13 | 26.72 |
|  | Arg |  | 29.81 ± 2.79 | 99.59 ± 0.09 | 68.39 | 22.72 |
|  | Tyr |  | 30.48 ± 4.82 | 99.67 ± 0.14 | 64.97 | 24.32 |
|  | Cho |  | 23.95 ± 1.29 | 99.33 ± 0.04 | 67.68 | 25.29 |
| Solution | T80 |  | 26.23 ± 2.77 | 99.22 ± 0.57 | 58.67 | 31.55 |
|  | T20 |  | 26.54 ± 3.59 | 99.41 ± 0.06 | 63.43 | 28.18 |
|  | Leu-H |  | 28.23 ± 4.07 | 100.9 ± 0.53 | 51.86 | 39.59 |
|  | OA |  | 30.34 ± 2.37 | 100.2 ± 0.74 | 65.82 | 23.23 |
|  | Gly |  | 28.06 ± 3.54 | 100.3 ± 1.11 | 65.77 | 26.22 |
|  | LP |  | 28.59 ± 3.45 | 98.79 ± 0.09 | 60.73 | 30.46 |
|  | S80 |  | 26.26 ± 3.72 | 99.69 ± 0.01 | 67.50 | 33.20 |
|  | P407 |  | 24.89 ± 4.57 | 99.02 ± 0.01 | 59.27 | 31.71 |
| Vapor | Bo |  | 25.57 ± 1.53 | 98.99 ± 0.03 | 52.52 | 36.91 |
|  | Me |  | 24.75 ± 2.79 | 99.19 ± 0.19 | 52.24 | 32.68 |
|  | Li |  | 27.89 ± 1.53 | 100.5 ± 0.70 | 62.31 | 29.12 |

**Figure S1** (A) Aerodynamic behavior of physical mixtures (PM), mixing Leu solid form at ratios of 0.1% and 2% (by weight relative to 1 g of LA) to obtain the DEX-PM. (B-D) Comparison of NGI deposition profiles of DEX DPIs prepared by blending DEX with LA coated under varying solvent (ethanol) volumes, Leu masses, and treatment times. Comparison of CI (E) and FPF (F) parameters of DEX DPIs prepared by blending DEX with different LA types before and after solution coating (*n* = 1).

**Table S2** The 5-HMF concentration of all powders (*n* = 1).

| Sample name | Various Leu-H mass (%, w/w) | m (mg) | Abs | C (μg/mL) |
| --- | --- | --- | --- | --- |
| LA | - | 50.43 | 0.002 | 0.06 |
| LC-LA  (new batch) | 0.05 | 50.19 | 0.047 | 0.43 |
| LC-LA  (6 months) | 0.001 | 50.47 | 0.201 | 1.70 |
|  | 0.01 | 50.16 | 0.162 | 1.38 |
|  | 0.05 | 49.26 | 0.177 | 1.50 |
|  | 0.2 | 50.11 | 0.184 | 1.56 |

**Table S3** The residual solvent concentration of all powders (*n* = 1).

| Sample name | Various volumes (μL/g) | C (%) |
| --- | --- | --- |
| LA | - | 0.05 |
| LC-LA | 20 | 0.18 |
|  | 50 | 0.47 |
|  | 100 | 0.40 |
|  | 200 | 0.42 |

**Figure S2** Comparison of the total peak intensity among three powders in TOF-SIMS images.

***Statistical analysis of particle morphological parameter evolution.*** As illustrated in Figure S3B, the probability density distributions of volume changes for both S-LA and NLC-LA groups were negatively skewed, indicating a general shrinkage trend. Notably, the NLC-LA group exhibited a more pronounced volume reduction compared to the S-LA group, suggesting a stronger physical erosion effect. While the overall geometry, as characterized by sphericity remained stable (Figure S3C), distinct evolution in surface textures was observed. The NLC-LA group showed a significant reduction in 3D Solidity (Figure S3D), with an active particle ratio of 62.80%, implying significant microstructural loosening compared to the S-LA group.

**Figure S3 Density distributions of particle morphological parameter variations for S-LA and NLC-LA groups.** (A) Categorical statistics of changes in aspect ratio, classified by a threshold of 1×10^-6^. (B) Change in volume (μm^3^), showing the distribution of particle size variations. (C) Change in Sphericity, illustrating the geometric stability of the particles. (D) Change in 3D solidity, reflecting the evolution of surface texture and compactness.

**Figure S4** Plasma concentration-time profiles of individual rats between inhal. DEX-LA (A-C) and inhal. DEX-NLC-LA (D-F) groups *in vivo.*

**Figure S5** Comparison of pharmacokinetic parameters (C_max_, T_max_ and T_1/2_) between inhal. DEX-LA and inhal. DEX-NLC-LA groups (*n* = 3).

**Table S4** Pharmacokinetic parameters of DEX via inhaling DEX-LA and DEX-NLC-LA (*n* = 3, mean ± SD).

| Parameters | inhal. DEX-LA (A) | inhal. DEX-NLC-LA  (B) | Ratio  (B/A) |
| --- | --- | --- | --- |
| AUC _(0-24 h)_ (ng/mL^*^h) | 442.9±98.84 | 613.6±231.7 | 1.5 |
| AUC _(0-∞)_ (ng/mL^*^h) | 493.9±100.8 | 703.6±248.1 | 1.4 |
| C_max_ (ng/mL) | 131.6±60.16 | 137.3±45.56 | 1.1 |
| T_max_ (h) | 0.75±0.43 | 0.19±0.05 | 0.3 |
| T_1/2_ (h) | 2.86±0.54 | 3.45±1.02 | 1.2 |
| MRT_(0-t)_ (h) | 3.25±0.43 | 3.37±0.19 | 1.0 |

**References:**

[1]	C. Kizilyaprak, J. Daraspe, B. M. Humbel, *Journal of microscopy* **2014**, *254* (3), 109, <https://doi.org/10.1111/jmi.12127>.

[2]	T. Faber, J. T. McConville, A. Lamprecht, *Journal of controlled release : official journal of the Controlled Release Society* **2024**, *366*, 312, <https://doi.org/10.1016/j.jconrel.2023.12.048>.

[3]	A. G. Marshall, S. M. Damo, A. Hinton, Jr., *Trends in biochemical sciences* **2023**, *48* (6), 585, <https://doi.org/10.1016/j.tibs.2023.02.005>.

[4]	K. L. House, L. Pan, D. M. O'Carroll, S. Xu, *European journal of oral sciences* **2022**, *130* (2), e12853, <https://doi.org/10.1111/eos.12853>.
